# Whole-Genome-Based *Helicobacter pylori* Geographic Surveillance: A Visualized and Expandable Webtool

**DOI:** 10.3389/fmicb.2021.687259

**Published:** 2021-08-02

**Authors:** Xiaosen Jiang, Zheng Xu, Tongda Zhang, Yuan Li, Wei Li, Hongdong Tan

**Affiliations:** ^1^BGI-Shenzhen, Shenzhen, China; ^2^BGI Education Center, University of Chinese Academy of Sciences, Shenzhen, China; ^3^College of Life Sciences, University of Chinese Academy of Sciences, Beijing, China; ^4^Shenzhen Key Laboratory of Unknown Pathogen Identification, BGI-Shenzhen, Shenzhen, China

**Keywords:** *Helicobacter pylori*, genomic, antibiotic-resistant, phylogenetic, webtool, whole-genome sequencing, genotyping

## Abstract

*Helicobacter pylori* exhibit specific geographic distributions that are related to clinical outcomes. Despite the high infection rate of *H. pylori* throughout the world, the genetic epidemiology surveillance of *H. pylori* still needs to be improved. This study used the single nucleotide polymorphisms (SNPs) profiling approach based on whole genome sequencing (WGS) to facilitate genomic population analyses of *H. pylori* and encourage the dissemination of microbial genotyping strategies worldwide. A total number of 1,211 public *H. pylori* genomes were downloaded and used to construct the typing tool, named *Hp*TT (*H. pylori* Typing Tool). Combined with the metadata, we developed two levels of genomic typing, including a continent-scale and a country scale that nested in the continent scale. Results showed that Asia was the largest isolate source in our dataset, while isolates from Europe and Oceania were comparatively more widespread. More specifically, Switzerland and Australia are the main sources of widespread isolates in their corresponding continents. To integrate all the typing information and enable researchers to compare their dataset against the existing global database easily and rapidly, a user-friendly website (https://db.cngb.org/HPTT/) was developed with both genomic typing tools and visualization tools. To further confirm the validity of the website, ten newly assembled genomes were downloaded and tested precisely located on the branch as we expected. In summary, the *H. pylori* typing tool (*Hp*TT) is a novel genomic epidemiological tool that can achieve high-resolution analysis of genomic typing and visualizing simultaneously, providing insights into the genetic population structure, evolution analysis, and epidemiological surveillance of *H. pylori*.

## Introduction

*Helicobacter pylori* are one of the most sophisticated colonizers in the world that infects more than half of the world’s population, ranging from infants to the elderly (Suerbaum and Michetti, 2002). It is a Gram-negative bacterium that normally colonizes the gastric mucosa of humans with about 10–20% infection result in diseases (Pohl et al., 2019; Attila et al., 2020). The typical diseases that have been reported include gastritis, peptic ulcer, mucosa-associated lymphoid tissue (MALT) lymphoma, and gastric cancer (Ernst and Gold, 2000). Globally speaking, the risks of disease and the incidence and mortality of gastric cancer were geographically different (Kodaman et al., 2014).

*H. pylori* display a distinguished mutation rate among bacterial pathogens due to the lack of genes that initiate classical methyl-directed mismatch repair (MMR) (Alm et al., 1999). The high mutation and recombination rate made *H. pylori* genomes have enormous plasticity, facilitating this pathogen and enabling it to perfectly adapt to its host (Kang and Blaser, 2006; Didelot et al., 2013). It has been reported that *H. pylori* in chronic infection could take place through vertical and familial transmission (Schwarz et al., 2008; Ailloud et al., 2019). In within-host evolution, the mutation rate could reach ∼30 single nucleotide polymorphisms (SNPs) per genome per year (Kennemann et al., 2011), compared to *Escherichia coli* at ∼1 SNP per genome per year (Reeves et al., 2011). Taking into account this occurrence and large recombination events, a simple and efficient way to define the geographical pattern and epidemiological surveillance of *H. pylori* is needed (Yamaoka, 2009; Jolley et al., 2018).

The transmission of *H. pylori* transmission is slow, taking place mostly within a household it does not tend to spread like a rapid epidemic (Didelot et al., 2013). Their phylogeny was based on MLST genes and later whole genomes revealed a population structure primarily reflecting early human migration events especially out of Africa 60,000 years ago but not recent spreading (Falush et al., 2003). The global population was split into hp groups, each of which is split into hsp subgroups in the agreed convention. The hpEastAsia includes hspEastAsia, hspMaori, and hspAmerind (Kawai et al., 2011; Montano et al., 2015; Thorell et al., 2017).

To describe the population structure of *H. pylori*, genetic typing methods such as single gene typing (e.g., *cagA*, *vacA*) were recorded in previous studies (Salama et al., 2007; Yamaoka, 2009), while seven-gene multi-locus sequence typing (MLST) became the dominant tool in the later stage due to its simple and rapid typing strategy, which covers genes including *atpA*, *efp*, *mutY*, *ppa*, *trpC*, *urel*, *yphC* that categorize *H. pylori* into different sequence types (STs) (Achtman et al., 1999). However, the resolution of seven-gene MLST was still low, which limited us to tracing the epidemiological origins of *H. pylori* strains (Banerji et al., 2020). Comparatively, SNP typing covers comprehensive core genes that can generate a matrix comprising concatenated SNPs and location information in the genome, which facilitated the newly sequenced genomes to be comparable by mapping and increase the typing resolution.

It has been found that 7-gene MLST are also linked to regional epidemics across the world. The 7-gene MLST typing method enables the regional specific recognition based on the defined STs, in which geographical pattern is linked with the different risks of clinical disease. For example, non-African and African lineage could be associated with different risks of gastric disease (Campbell et al., 2001). Thus, geographic patterns can somehow link to the possibility of clinical disease. However, the seven-gene genotypes of *H. pylori* are diverse due to the high variability of *H. pylori* genomes, which hinders the recognition of patterns directly from the sequence types (STs) in 7-gene MLST. In addition, there is no information on geographical patterns or visualization tools for seven-gene MLST, thus such related geographic patterns were hard to find when a new ST was found.

This study describes a *H. pylori* genomic typing tool, *Hp*TT (*H. pylori* Typing Tool) that uses the SNP profiling based on whole-genome sequencing data. In addition to genomic typing, *Hp*TT also provides a phylogenetic and geographic visualization tool based on the Nextstrain framework (Hadfield et al., 2018). This tool allows users to upload *H. pylori* WGS data for genomic typing and uncover possible transmission events of *H. pylori.* It is believed that this tool can not only improve genome typing resolutions but may also predict the possible origin of the epidemic *H. pylori* isolates, enabling the global surveillance of *H. pylori*.

## Materials and Methods

### *Helicobacter pylori* Genomes Downloaded and Filtered in This Study

A total number of 1,654 assembled *H. pylori* genomes were downloaded from the NCBI RefSeq database (genomes available as of May 4, 2020) using the ncbi-genome-download tool (version 0.2.12). The corresponding metadata of assembled genomes was searched by function using Entrez Direct (version 10.9) (Kans, 2020). By metadata filtering, 1,211 genomes were selected with sample collection location available (Table 1). All genomes were scanned by mlst (version 2.11) with the library of MLST updated on December 31, 2020 (Jolley and Maiden, 2010).

**TABLE 1:** Summary of 1,211 *H. pylor*i genomes.

Continent	Country (region) of origin	Number of isolates
Asia		312 (25.76%)
	Cambodia	53
	China	74
	China (Taiwan)	8
	India	47
	Indonesia	1
	Japan	31
	Kuwait	2
	Malaysia	79
	North Korea	1
	Singapore	14
	South Korea	1
	Vietnam	1
Africa		10 (0.82%)
	Morocco	6
	Nigeria	1
	South Africa	3
Europe		294 (24.28%)
	Belarus	2
	Belgium	6
	France	37
	Germany	31
	Ireland	1
	Poland	2
	Portugal	1
	Russia	3
	Spain	54
	Sweden	19
	Switzerland	130
	United Kingdom	8
Oceania		178 (14.70%)
	Australia	177
	Papua New Guinea	1
North America		233 (19.24%)
	Canada	2
	El Salvador	1
	Mexico	118
	Nicaragua	24
	United States of America	88
South America		184 (15.19%)
	Angola	1
	Colombia	172
	Peru	11

### SNP Analysis

The 1,211 assembled genomes were mapped to the reference genome *H. pylori* 26695 (GenBank: AE000511.1) (Tomb et al., 1997) using MUMmer (version 3.23) (Kurtz et al., 2004). SNPs were filtered with a minimum mapping quality cutoff at 0.90 across 1,211 assembled *H. pylori* genomes. 6,129 SNPs were found, and an SNP profile of *H. pylori* is established for the corresponding isolates.

### Phylogenetic Analysis

The maximum likelihood (ML) phylogenetic tree was constructed by iqtree (version 2.0.3) (Nguyen et al., 2015) based on 6,129 SNPs alignments of all 1,211 isolates. The reference genome *H. pylori* 26695 was used as an outgroup. The tree was generalized by the Gamma distribution to model site-specific rate variation (the GTR model). Bootstrap pseudo-analyses of the alignment were set at > = 1000. All ML trees were visualized and annotated using Figtree (version 1.4.4). The minimum spanning tree was constructed by the GrapeTree (v1.5.0) (Zhou et al., 2018). The mutation rate of the *cagA* gene was calculated by BEAST v1.8.4 (Suchard et al., 2018).

### Geographic Typing System

Based on the phylogenetic tree, two levels of the geographic group were defined, including the first level defined at the continent scale and the second level defined as a country-specific scale. In the first level of genotyping, lineages carrying more than seven isolates and >75% isolates sourced from one major continent were defined as a continent-specific group or clade. A mixed continent group was defined when there was no major continent identified with isolates at >75%. In the second level, lineages carrying more than one isolate and >75% isolates sourced from one major country were defined as a country-specific group or subclade. In addition, a mixed group was also defined at level two when there were more than two isolates and not a major country identified with isolates at >75%. The association of the genomic lineage of *H. pylori* with the geographic information of isolates provided a map that allows us to trace both the possible transmission and evolution of a detected or sequenced *H. pylori* genome.

### Establishment of *Helicobacter pylori* Database

The *Hp*TT website was established based on two modules: (1) The genomic-geographical typing tool of *H. pylori* isolates and (2) a visualization tool of both the genomic and geographic typing results. The online typing tool was written in PHP, Javascript, css, and HTML. The online visualization service was performed based on the CodeIgniter framework^1^, tree visualization was analyzed by the augur^2^ bioinformatics tool and the auspice^3^ visualization tool imbedded in the Nextstrain (Hadfield et al., 2018) open source project. The *H. pylori* database was stored in a Mysql database.

## Results

### Definition of Two Levels of Geographic Genotypes for *Helicobacter pylori*

**FIGURE 1:**
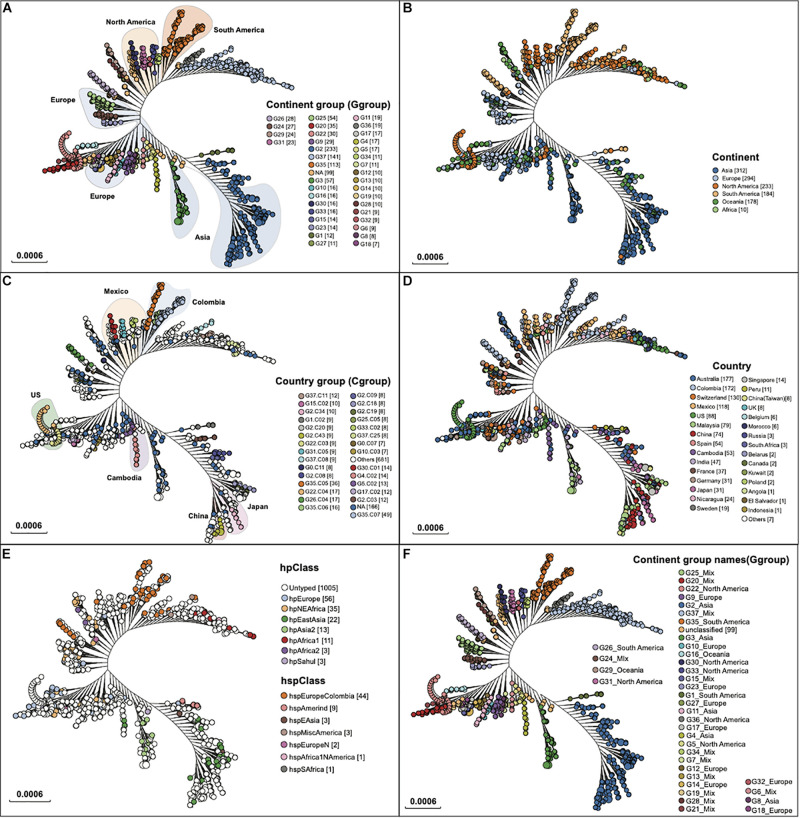
Two clades of geographic typing based on the WGS. The *Hp*TT enrolled 1,211 *H. pylori* genomes downloaded from NCBI. The clade nodes in each figure correspond to **(A)** G groups for continent level of typing, **(B)** the continent that isolates collected from, **(C)** C groups for country-level typing, **(D)** the country that isolates were collected from. **(E)** the hp Class and hsp Class, **(F)** G groups for continent level of typing with group names. Numbers in parenthesis refer to the number of isolates in each genogroup.

A total of 1,211 assembled genomes with available geographic information from the NCBI RefSeq database were downloaded and analyzed for establishing the *H. pylori* genotyping database (Supplementary Table 1). All assembly genomes were mapped to the reference genome *H. pylori* 26695. Based on the maximum likelihood tree, 6,129 SNPs extracted from 1,135 genes on the reference genome were defined for further genomic typing. In terms of geographic information, 1,112 isolates were grouped at two levels, including 37 continent-level groups (Figures 1A,B) and/or 236 country-level groups (Figures 1C,D). The median pairwise distances (the median number of SNPs shared by the branches) between isolates were found as follows: 319 SNPs within continent clades and 1,493 SNPs within country subclades. We labeled these continent clades and country subclades using a structured hierarchical nomenclature system similar to that used for *M. tuberculosis* (Coll et al., 2014). For instance, region 1 clade (G1) is subdivided into country subclades G1.C1 and G1.C2. The mutation rate of *cagA* was 2.413 × 10^–2^ (95% CI: 1.600 × 10^–2^–3.900 × 10^–2^), which was 1.739 × 10^–2^/site/year (95% CI: 1.153 × 10^–2^–2.811 × 10^–2^).

### A Continent Level Genomic Typing for *Helicobacter pylori*

A total number of 37 continent level groups (*n* = 1,112) were defined, including 25 continent-specific groups and 12 mixed-continent groups (Figures 1A,B). Isolates across the tree did not fall into the continent group but can be defined as a country group that was named G0 (*n* = 74). Isolates across the tree that fell into neither fall into the country group nor the continent group were defined as non-grouped (*n* = 25). Because the genome data of *H. pylori* were downloaded from the NCBI database, and these genomes came from various regions of the world. Compared with their ancestors, these strains have different genomes, which has led to the formation of independent evolutionary branches. After they formed independent evolutionary branches, (1) they may not have spread. (2) After the spread, it was not collected. These two reasons could account for an insufficient number of strains in the branch, which cannot form a group with regional characteristics under our typing method.

**FIGURE 2:**
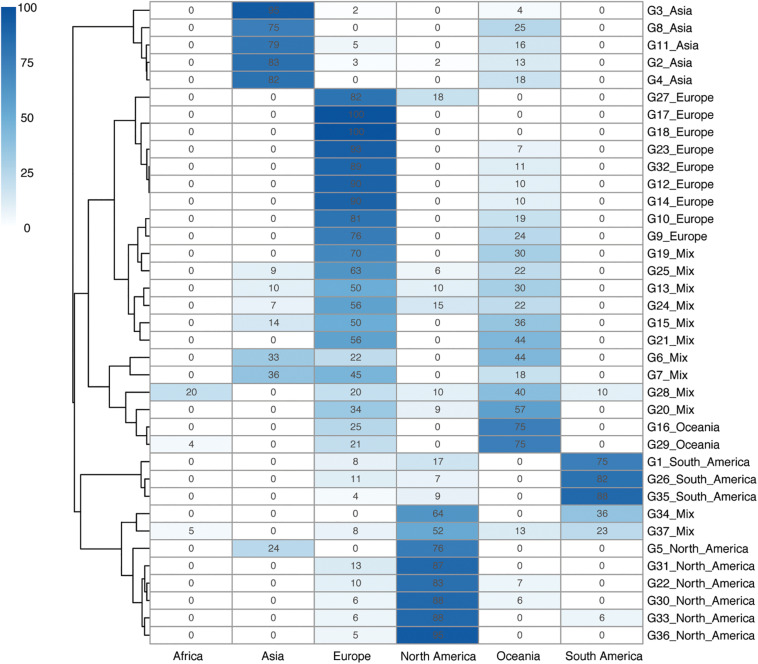
Geographical clustering of *H. pylori* continent clades. The number in each cube represents the percentage of unique isolates sourced from each of the continents. A total number of 37 continent-level groups were defined. The deeper the color, the higher the percentage of the isolates in that continent level of clade groups. A phylogenetic tree is also shown on the left side of the table. Background information on the isolates is provided in Supplementary Table 1.

Five continent-specific groups contain more than 75% Asian isolates, supporting Asia to be the continent with the largest isolate source (*n* = 319, 26.34%) (Figure 2). North America was found to be the second-largest group of isolate pool which consisted of six continent-specific groups (*n* = 132, 10.90%). Although fewer isolates were found to be sourced from Europe (*n* = 109, 9.00%), these isolates were distributed in nine continent-specific groups. Two groups (G16 & G29) of isolates were found to be part of the Oceania specific group (*n* = 39, 3.22%) and three groups (G1 & G26 & G35) were found to be from the South America specific groups (*n* = 109, 9.00%). In addition, the 12 mixed groups of isolates contained 226 isolates (18.66%). Among all G level groups, G2 was the largest continent specific group (*n* = 223) that mainly contained isolates from Asia (193/223, 82.83%), while G35 was the second largest continent specific group (*n* = 109) that mainly contained isolates from South America (99/109, 87.61%). Apart from all the continent groups above, there was no Africa-specific group found, but only with isolates collected from Africa defined in G28 (*n* = 2), G37 (*n* = 7), and G29 (*n* = 1) (Figure 2).

Although the continent-specific groups did not 100% stick to one continent in our typing system, the transmission events were still possible to predict. While most of the Asian isolates fell into the Asia groups, a small proportion of the Asian isolates belonged to the mixed groups. Similarly, most of the isolates sourced from North America and South America fell in their own region groups, while a minority of the isolates were in the mixed groups. Interestingly, isolates from Oceania and Europe could be found across all 12 mixed continent groups, reflecting the fact that *H. pylori* isolates from these two continents were relatively widespread across the globe.

### The Nested Country Level Genomic Typing for *Helicobacter pylori*

**FIGURE 3:**
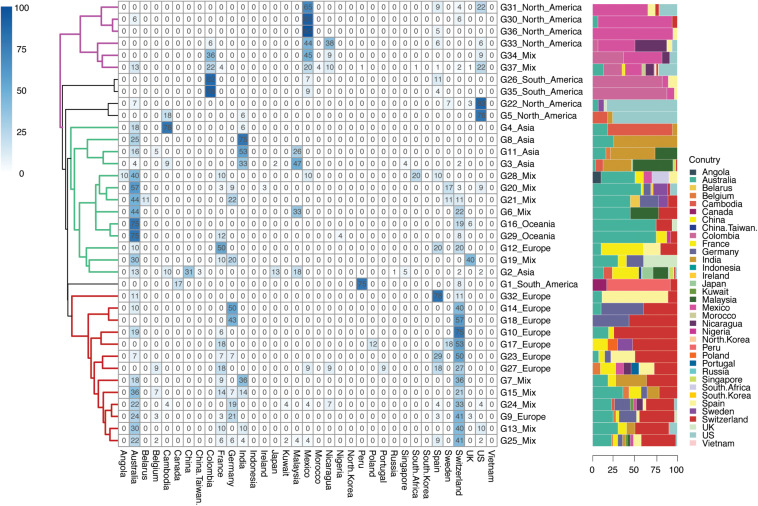
Geographical clustering of *H. pylori* country subclades. The number in each cube represents the percentage of unique isolates sourced from each of the countries in that continent group. A total number of 216 country-level groups were defined. The deeper the color, the higher the percentage of the isolates sourced from that country in continent-level groups. Background information on isolates is provided in Supplementary Table 1.

A total number of 859 isolates were grouped into 216 geographic patterns at a country level, which were predominant in 29 countries across six continents (Figure 3). Among these 29 countries encompassing 216 groups, 20 countries found in 168 groups were defined as country-specific groups, while the remaining 9 countries were scattered over the 48 country-level mixed groups that were left.

G35.C07 was the largest country-specific group that contained 49 isolates from Colombia, followed by the G35.C05 (*n* = 35) dominated in Colombia as well. These isolates from Colombia were mainly collected from the NCBI Bioproject PRJNA352848, which study contained the population structure of *H. pylori* in regional evolution in South America (Muñoz-Ramírez et al., 2017). The isolates from groups G35.C07 and G35.C05 were mainly found in Colombia, Mexico, and Spain (Figure 3). This result provided evidence that the *H. pylori* isolates were possibly transmitted from Spain and spread locally in South America and North America. In comparison, Australia and Switzerland were the largest countries of isolate sources with isolates scattered across more than half of the country-specific groups.

When comparing the percentage of isolates from different countries, those isolates from France, Germany, Malaysia, Nicaragua, Sweden, and the United Kingdom were found to be scattered in more than one continent group, while isolates from Cambodia, Colombia, India, Peru, Spain, and the United States were focused in one continent group when they were also found in other continent groups. More importantly, Australia and Switzerland were two countries that were mostly found to have scattered isolates in different regional specific groups.

Three clusters were observed in the percentage of different isolate sources at continent scale (G32 to G25 with red branches in Figure 3), consisting of groups from Europe and mixed continents. Specifically, those isolates from mixed groups were mainly sourced from European and Oceania countries, making this cluster dominated by Europe-Oceania. The second cluster was the mixed by Asian, Oceanian, European, and mixed groups (G4 to G2 with green branches in Figure 3) but dominated by isolates from Australia and Asian countries. Therefore, cluster two was specified as the Asian-Pacific cluster. The third cluster was formed by North American groups (G31 to G37 with purple branches in Figure 3), while South American branches were next to the North American cluster.

### Comparing With hp and hsp Class

hp and hsp class were designed for the geographic-genetic typing of *H. pylori* (Kawai et al., 2011; Montano et al., 2015; Thorell et al., 2017; Lamichhane et al., 2020). Of 1,211 *H. pylori* genomes, 231 were found to have been typed by hp and hsp class, which were well fit to our typing groups. Specifically, hpEastAsia, hpAsia2, and hspEAsia were included in the three Asia continent groups G2, G3, and G4 (Figures 1E,F), while hspEuropeColombia fell in two south America groups, G26 and G35. Similarly, hpAfrica1, hspMiscAmerica, and hspAfrica1NAmerica were mapped to a mixed group G37. The comparison with hp and hsp clusters enhanced the validity of our typing method.

### Comparing With Seven-Gene MLST

Seven-gene MLST was implied to get the sequence types (STs) for all 1,211 isolates. Unfortunately, due to the high mutation rate of the *H. pylori* strains, most of the seven-gene allele were only found to have high similarity instead of an accurate type, as a result, a large number of isolates (*n* = 876, 72.3%) were untyped in our dataset (Supplementary Table 1 and Figure 1). Among all the countries, Australia and Switzerland were the two countries with a higher number of untyped isolates, which is probably due to the isolates being collected by those two countries having not been submitted to the pubMLST website to be typed.

### A User-Friendly Typing Website

**FIGURE 4:**
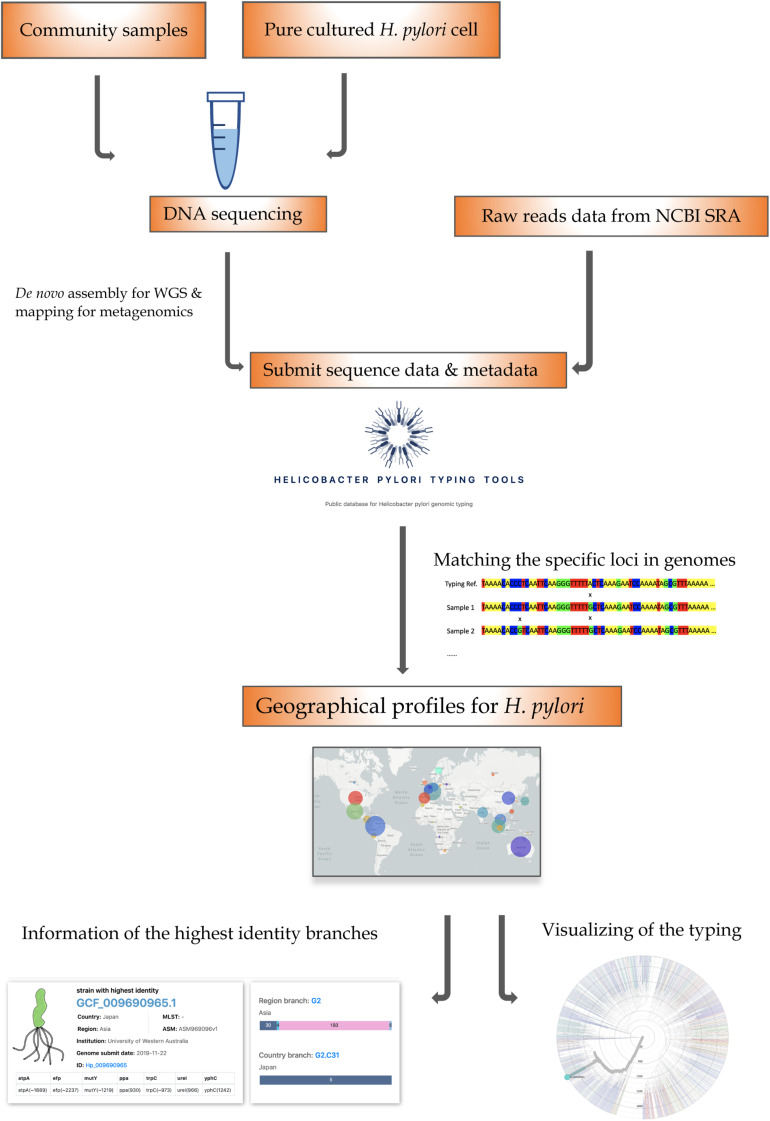
The *Hp*TT workflow. The SNP-based genotyping approach can be used with the Whole Genome Sequencing (WGS) data, which can be acquired in the following ways: DNA can be extracted from a pure cultured bacterial cell with WGS data or a community sample with metagenomic sequencing data. After being sequenced by an appropriate platform, the assembled genomes can be directly submitted to our database. In addition, the public assembled data can also be directly submitted to our database. The downstream analyses of the aligned sequence data can be linked to the phylogenetic and geographic page.

To support our *H. pylori* geographic typing tool, a user-friendly typing website was established and made available at https://db.cngb.org/HPTT/. Our *Hp*TT approach is compatible with any whole-genome sequencing (WGS) data with metadata (Figure 4). For the sequencing data from pure-cultured isolates, the assembled genomes can be directly submitted to our website. However, it is worth noting that sequences or assembled genomes needed to be extracted from metagenome samples before submission (Parks et al., 2017; Olekhnovich et al., 2019). Except for the sequenced genome data, the available assembled contigs from NCBI Sequence Read Archive (SRA) or assembly database (RefSeq), or other genome databases (e.g., European Nucleotide Achieve) can also be directly uploaded to our website. By using MUMmer alignment and blast process, the uploaded genome can be located to the closest matching genomes, further facilitating the possible transmission route analysis across the globe. In addition, our database can be also linked to the NCBI genome database, helping the user easily locate the metadata information from the available database (see Supplementary Material).

Except for the typing tool, the Nextstrain framework was also embedded in our website. By clicking the uploaded genome number, information can be linked to the phylogenetic tree with the corresponding continent and country. Possible evolution relationships and interactive located functions have made our typing tools easy to be applied and understood.

### The Validation of Our Genomic Typing Method

For validating the accuracy of the genomic typing method and the efficiency of the web tool, ten new genomes from NCBI were downloaded and tested (Supplementary Table 2). Except for one genome (GCF_002206465.1), which failed due to being sequenced by Pacbio, the remaining nine genomes were typed successfully [Our typing tool was established based on the MPS (Massive Parallel Sequencing) data, Pacbio sequencing may generate many SNPs in the gap region in MPS sequencing].

## Discussion

The epidemiological patterns of *H. pylori* isolates have been reported with specific geographic characteristics. In this study, the new typing webtool *Hp*TT not only illustrated the population structure of *H. pylori* but also made genomic typing easy to approach. In the continent level of typing, 1,112 isolates were grouped into 37 continent-specific patterns. Except for 12 continent mixed groups, the rest could be defined as continent-specific groups across the five continents. Isolates from Europe and Oceania were universally found in most of the continent-level groups (Europe 33/37, 89.19% and Oceania 26/37, 70.27%), illustrating that isolates from these two continents were widely spread across the world.

In the country level of typing, 1,045 isolates were grouped into 216 country-level groups. Most of the isolates were defined as country-specific groups (168/216, 77.77%), while the rest of the isolates were grouped as country mixed groups (48/216, 22.22%). Australian and Swiss isolates were found to be widespread around the world, while isolates from Columbia were more regionally specific. It has been reported that *H. pylori* in South America were originally transmitted from Spain (Muñoz-Ramírez et al., 2017), this data perfectly aligned with our results in G35.C05 and G35.C07, giving support to the accuracy of our genomic typing method.

The phylogenetic tree in this study was built by the collection of *H. pylori* genomes downloaded from the NCBI Refseq database. Ideally, all the isolates would be able to be grouped into different geographic groups, but there are still a few isolates that cannot be grouped by our typing tool due to the following reasons: (1) They have not spread after forming independent evolutionary branches, (2) After spreading, their offspring have not been collected and sequenced.

*H. pylori* show high and fine (∼40 bp patch) intergenic recombination (Bubendorfer et al., 2016), which leads to sharing patches of genome sequences and makes the phylogenetic relationship obscure. Special methods have been developed to infer a population structure based on this sharing (Yahara et al., 2013). Although such typing methods are built based on core SNPs that cannot accurately trace the origin of the isolates comparing to a recent comprehensive study of *H. pylori* (Muñoz-Ramírez et al., 2021), we established a simple, rapid, and user-friendly genetic-geographic typing tool in the population structure description. The core SNPs of 1,211 *H. pylori* genomes were filtered with a minimum mapping quality cut off at 0.90, which means the individual indels for isolates were not kept. Our typing method has been further validated by testing genomes, suggesting that the typing tool was successfully established.

The addition of 7-gene MLST to our database intended to offer an easy way for users to visualize both results from our typing method and 7-gene MLST with comparisons. The large set of untyped isolates in 7-gene MLST might be related to the insufficient submission of genomes to pubMLST. In our typing database, isolates collected from Australia and Switzerland were scattered across different regional groups, which might be due to the frequent transmission event that occurred between Australia/Switzerland and other countries.

In this study, except for the novel typing tool, a user-friendly website was also established. By using this typing tool, users can achieve fast and precise genomic typing, easily locating the possible origins and transmission events across the world. When located in the actual geographic group, it is easy for users to check the details of the corresponding components of the branches in our database. The genome with the highest identity can be easily linked to the NCBI database as well as the visualization tool where the dynamic evolution of *H. pylori* was shown. At the same time, seven-gene MLST results were displayed for each genome in the database, as well as the hp groups and hsp subgroup results studied previously (Kawai et al., 2011; Yahara et al., 2013).

The most interesting part of the *Hp*TT tool and methodology allows us to perform genome typing with assembled genomes from the metagenomics samples, as illustrated in Figure 4. Due to the rapid mutation of *H. pylori*, it is most likely that the sample from one’s gut is heterogeneous. Whole-genome sequencing by combining sequencing libraries labeled with different barcodes on a meta sample, and a cultured pure isolate could yield enough data from one single run to perform the epidemiological surveillance of *H. pylori* on a global level to find the possible transmission event in evolution profile. An open-source assay protocol will be developed and shared in the future to combine with this *Hp*TT tool to enable the epidemiological surveillance of *H. pylori*.

Although our typing tool filled a gap in the genetic epidemiological surveillance of *H. pylori*, some functions still need to be improved. For example, cytotoxin-associated gene A (*cagA*) and *vacA* were two crucial genes that were reported to be correlated with geographic patterns of *H. pylori* (Yamaoka, 2009; Breurec et al., 2011). The *cagA* gene is one of the most important virulence genes in *H. pylori*, located at the end of a cag pathogenicity island (cag PAI) that encodes 120–145 kDa CagA protein (Šterbenc et al., 2019). Another virulence factor was vacuolating cytotoxin encoded by the gene *vacA* (Šterbenc et al., 2019). The variation of these two genes was widely reported by the *H. pylori* groups that can reflect the genomic difference for different geographic patterns. However, such a rapid typing method on a website for these two genes is still lacking, which could be considered in the further *Hp*TT version 2.

*H. pylori* are normally treated by antibiotics without antimicrobial susceptibility testing (Pohl et al., 2019). Antibiotics-resistant *H. pylori* has been reported related to several mutations within the genes *pbp1A*, *23S rRNA*, *gyrA*, *rdxA*, *frxA*, and *rpoB* (Domanovich-Asor et al., 2021). These antibiotics-resistant genes will be included in the second version despite there already being an antibiotics-specific resource available (Yusibova et al., 2020). As more strains or isolates are being deposited into our database along with geographic information, *Hp*TT could be more powerfully associate genomic typing with geographic information and phenotypes.

In summary, this work illustrates efforts in a global epidemiological study of *H. pylori* isolates. Two functions were designed for the web typing tool, one for genomic typing and the other for phylogenetic and geographic visualization. The accuracy of our genomic typing system was proved by ten unused genomes as well as in another published study (Muñoz-Ramírez et al., 2017). Together with the visualization tool, the genomic population structure of *H. pylori* with geographic documents were described. Future studies will be expanded by the crucial virulence gene and antibiotic-related genes. This tool is beneficial for the surveillance of *H. pylori* for public health and the monitoring of its epidemic development.

## Data Availability Statement

All assembled *H. pylori* genomes used in this study were downloaded from NCBI assembly database (https://www.ncbi.nlm.nih.gov/assembly/) under the accession numbers in Supplementary Tables 1, 2.

## Author Contributions

ZX and HT conceived the study. XJ performed the analysis. TZ, YL and WL revised the manuscript. HT provided critical analysis and discussions. All authors discussed the results and contributed to the revision of the final manuscript.

## Conflict of Interest

The authors declare that the research was conducted in the absence of any commercial or financial relationships that could be construed as a potential conflict of interest.

## Publisher’s Note

All claims expressed in this article are solely those of the authors and do not necessarily represent those of their affiliated organizations, or those of the publisher, the editors and the reviewers. Any product that may be evaluated in this article, or claim that may be made by its manufacturer, is not guaranteed or endorsed by the publisher.

## Footnotes

1
https://www.codeigniter.com/

2
https://github.com/nextstrain/augur

3
https://github.com/nextstrain/auspice
