# Supplementary figures and images for: Whole-Genome-Based Helicobacter pylori Geographic Surveillance: A Visualized and Expandable Webtool
Source: Front Microbiol. 2021 Aug 2;12:687259. doi: 10.3389/fmicb.2021.687259 (PMC8366602; doi:10.3389/fmicb.2021.687259)

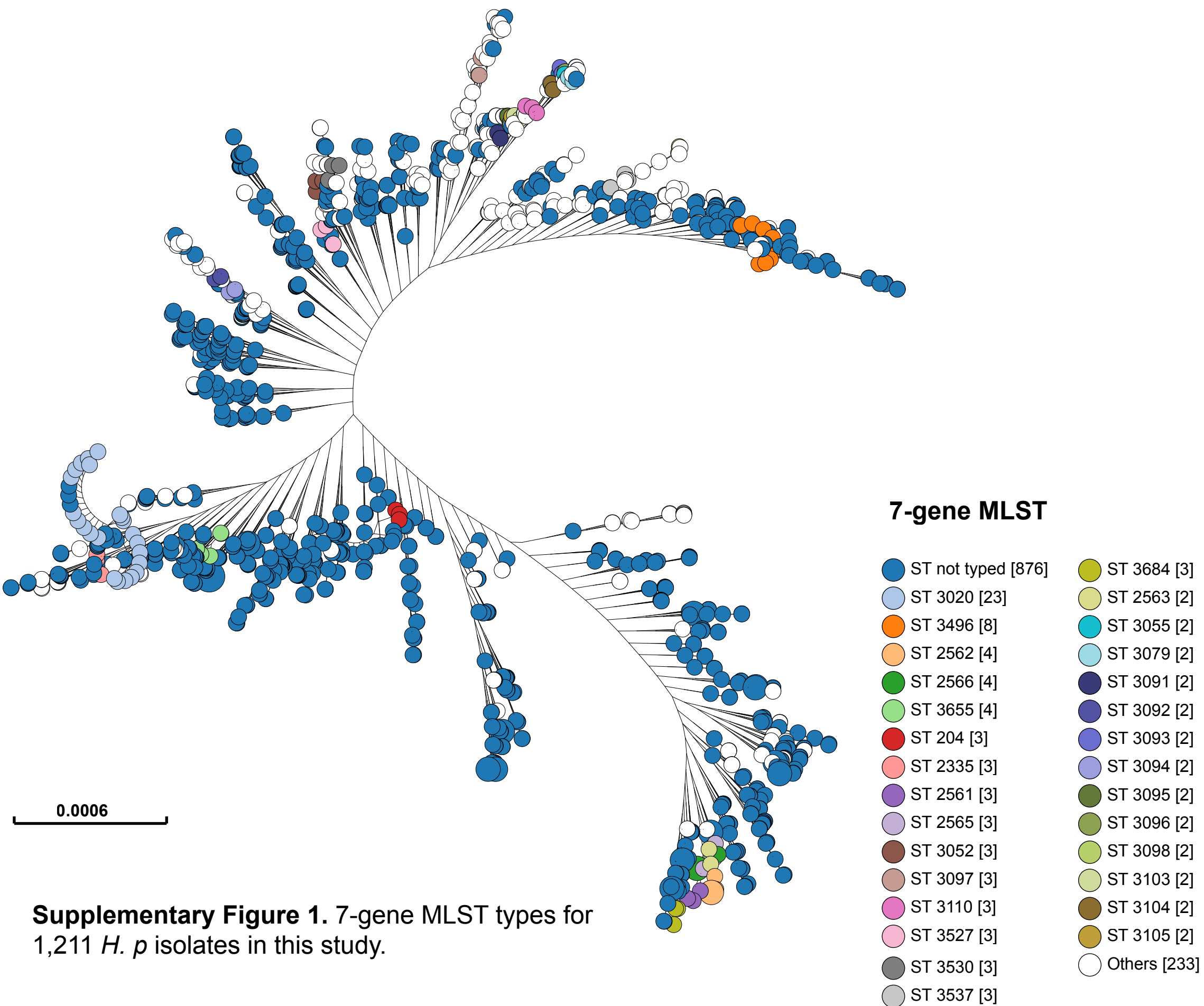

**Supplementary Figure 1.** 7-gene MLST types for 1,211 *H. p* isolates in this study.

Supplement: Supplementary file 1 [file Image_1.pdf]
