# Supplementary material for: Whole-Genome-Based Helicobacter pylori Geographic Surveillance: A Visualized and Expandable Webtool
Source: Front Microbiol. 2021 Aug 2;12:687259. doi: 10.3389/fmicb.2021.687259 (PMC8366602; doi:10.3389/fmicb.2021.687259)
Supplement: Supplementary file 2 [file Data_Sheet_1.PDF]

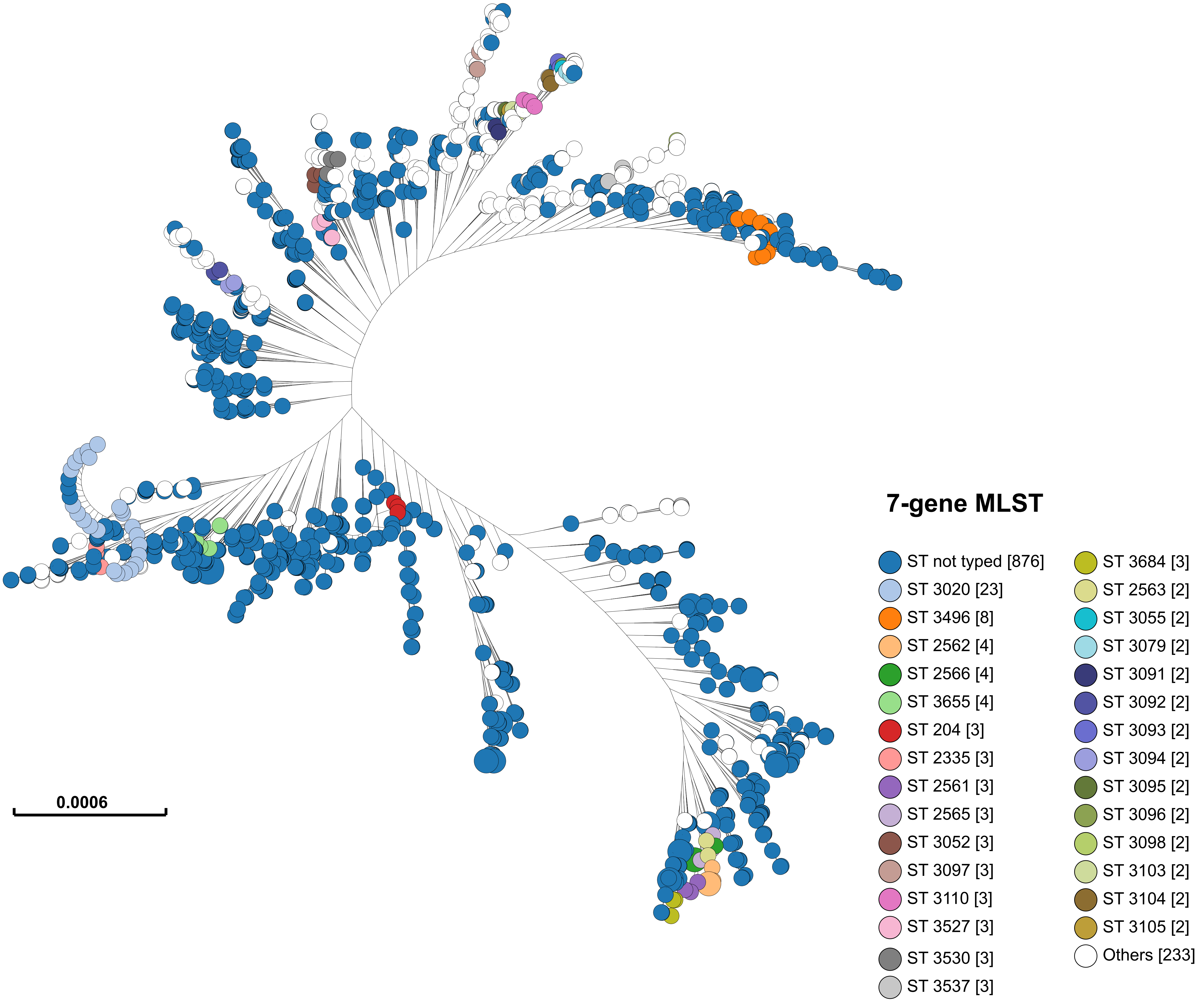
7-gene MLST
ST not typed [876]
ST 3020 [23]
ST 3496 [8]
ST 2562 [4]
ST 2566 [4]
ST 3655 [4]
ST 204 [3]
ST 2335 [3]
ST 2561 [3]
ST 2565 [3]
ST 3052 [3]
ST 3097 [3]
ST 3110 [3]
ST 3527 [3]
ST 3530 [3]
ST 3537 [3]
ST 3684 [3]
ST 2563 [2]
ST 3055 [2]
ST 3079 [2]
ST 3091 [2]
ST 3092 [2]
ST 3093 [2]
ST 3094 [2]
ST 3095 [2]
ST 3096 [2]
ST 3098 [2]
ST 3103 [2]
ST 3104 [2]
ST 3105 [2]
Others [233]
0.0006
