# Supplementary material for: Whole-Genome-Based Helicobacter pylori Geographic Surveillance: A Visualized and Expandable Webtool
Source: Front Microbiol. 2021 Aug 2;12:687259. doi: 10.3389/fmicb.2021.687259 (PMC8366602; doi:10.3389/fmicb.2021.687259)
Supplement: Supplementary file 3 [file Data_Sheet_2.PDF]

Supplementary Table 1. Strains information and typing results

No._Of_Strains	Accession number	Continent	Country	Ggroup	Cgroup	MLST types
Hp_002357575	GCF_002357575_1	Asia	Japan	G2	G2.C34	-
Hp_003639245	GCF_003639245_1	Europe	Spain	G25	G25.C06	-
Hp_002012505	GCF_002012505_1	South America	Colombia	G35	G35.C07	3103
Hp_000285895	GCF_000285895_1	Europe	France	G17	G17.C01	3655
Hp_003639785	GCF_003639785_1	Europe	Spain	G32	G32.C01	-
Hp_001542505	GCF_001542505_2	Asia	Malaysia	G2	G2.C40	-
Hp_003635825	GCF_003635825_1	Oceania	Australia	G25	G25.C05	-
Hp_002533065	GCF_002533065_1	South America	Colombia	G26	G26.C04	3143
Hp_000685705	GCF_000685705_1	Oceania	Australia	G20	NA	-
Hp_000499345	GCF_000499345_1	Europe	Russia	G2	G2.C01	-
Hp_003637925	GCF_003637925_1	Oceania	Australia	G25	NA	-
Hp_004012975	GCF_004012975_1	Europe	Switzerland	NA	0.C12	-
Hp_009737175	GCF_009737175_1	North America	United States of America	G20	G20.C03	-
Hp_001275165	GCF_001275165_1	Asia	Malaysia	G2	G2.C24	-
Hp_009741105	GCF_009741105_1	North America	United States of America	G37	NA	-
Hp_002092915	GCF_002092915_1	Africa	Morocco	G37	G37.C25	3496
Hp_006439905	GCF_006439905_1	Asia	Cambodia	NA	0.C02	-
Hp_003638105	GCF_003638105_1	Oceania	Australia	G20	G20.C07	-
Hp_011141015	GCF_011141015_1	North America	Mexico	G37	G37.C11	-
Hp_003638625	GCF_003638625_1	Oceania	Australia	G20	G20.C04	1144
Hp_009690305	GCF_009690305_1	Asia	China	G2	G2.C04	-
Hp_002012395	GCF_002012395_1	South America	Colombia	G35	G35.C07	3104
Hp_011140875	GCF_011140875_1	North America	Mexico	NA	NA	-
Hp_003637485	GCF_003637485_1	Oceania	Australia	G28	NA	-
Hp_003636005	GCF_003636005_1	Oceania	Australia	G2	G2.C09	-
Hp_009691005	GCF_009691005_1	Asia	China	G2	G2.C20	-
Hp_003637345	GCF_003637345_1	Oceania	Australia	G2	G2.C06	-
Hp_003638285	GCF_003638285_1	Oceania	Australia	G19	G19.C03	1445
Hp_003939085	GCF_003939085_1	Europe	Belarus	G21	NA	-
Hp_003060065	GCF_003060065_1	Europe	France	NA	0.C08	-
Hp_000401335	GCF_000401335_1	Asia	Malaysia	G3	G3.C03	-
Hp_002081955	GCF_002081955_1	Africa	Morocco	G37	G37.C25	3496
Hp_003058635	GCF_003058635_1	Europe	Sweden	G17	G17.C02	-
Hp_002012715	GCF_002012715_1	South America	Colombia	G35	G35.C07	3108
Hp_000685625	GCF_000685625_1	North America	United States of America	G5	G5.C02	-
Hp_004013875	GCF_004013875_1	Europe	Switzerland	G25	NA	-
Hp_002012935	GCF_002012935_1	South America	Colombia	G26	G26.C04	3129
Hp_004012215	GCF_004012215_1	Europe	Switzerland	G9	NA	-
Hp_011140795	GCF_011140795_1	North America	Mexico	NA	0.C06	-
Hp_002832045	GCF_002832045_1	Asia	India	G8	G8.C02	-
Hp_003637165	GCF_003637165_1	Oceania	Australia	G16	G16.C04	-
Hp_002952175	GCF_002952175_1	North America	United States of America	G22	G22.C04	-
Hp_006440345	GCF_006440345_1	Asia	Cambodia	G3	G3.C02	-
Hp_009690255	GCF_009690255_1	Asia	China	G2	G2.C19	-
Hp_000591295	GCF_000591295_1	Asia	China	G2	G2.C42	2565
Hp_002832195	GCF_002832195_1	Asia	India	G11	G11.C03	-
Hp_002952235	GCF_002952235_1	North America	United States of America	G22	G22.C04	3020
Hp_004013315	GCF_004013315_1	Europe	Switzerland	G13	G13.C02	-
Hp_009690015	GCF_009690015_1	Asia	China	G2	G2.C30	-
Hp_000685745	GCF_000685745_1	Oceania	Australia	G13	G13.C01	-
Hp_002357655	GCF_002357655_1	Asia	Japan	G2	G2.C34	-
Hp_004014535	GCF_004014535_1	Europe	Switzerland	G18	G18.C02	-
Hp_009741095	GCF_009741095_1	North America	United States of America	G31	G31.C03	-
Hp_002026585	GCF_002026585_1	North America	Mexico	G34	G34.C03	3520
Hp_009690345	GCF_009690345_1	Asia	China	G2	G2.C36	-
Hp_004013635	GCF_004013635_1	Europe	Switzerland	G10	G10.C01	-
Hp_009689925	GCF_009689925_1	Asia	China	G2	G2.C32	-
Hp_004088155	GCF_004088155_1	Asia	China	G2	G2.C07	-
Hp_004013195	GCF_004013195_1	Europe	Switzerland	G23	G23.C02	-
Hp_009741145	GCF_009741145_1	North America	United States of America	G2	G2.C49	-
Hp_001185035	GCF_001185035_1	Asia	Malaysia	G3	NA	-
Hp_006339065	GCF_006339065_1	Europe	Germany	G21	NA	-
Hp_009689765	GCF_009689765_1	Asia	China	G2	G2.C10	-
Hp_000689095	GCF_000689095_1	North America	United States of America	G5	G5.C02	-
Hp_003635785	GCF_003635785_1	Oceania	Australia	G37	G37.C18	-
Hp_006438425	GCF_006438425_1	Asia	Cambodia	G2	NA	-
Hp_003637305	GCF_003637305_1	Oceania	Australia	G2	NA	-
Hp_000448505	GCF_000448505_1	South America	Colombia	G35	G35.C06	-
Hp_003059085	GCF_003059085_1	Europe	Sweden	G21	NA	-
Hp_003639855	GCF_003639855_1	Europe	Spain	NA	0.C07	-
Hp_003635665	GCF_003635665_1	Oceania	Australia	G37	G37.C33	-
Hp_000470075	GCF_000470075_1	Europe	Germany	G14	G14.C02	-
Hp_003382505	GCF_003382505_1	Europe	Spain	G23	G23.C04	-
Hp_003635945	GCF_003635945_1	Oceania	Australia	G2	NA	-
Hp_901687255	GCF_901687255_1	Oceania	Australia	G9	G9.C04	-
Hp_003059615	GCF_003059615_1	Europe	France	G37	G37.C32	-
Hp_006339235	GCF_006339235_1	Europe	Germany	G25	G25.C11	-
Hp_009689685	GCF_009689685_1	Asia	China	G2	G2.C37	-
Hp_011175325	GCF_011175325_1	North America	Nicaragua	G37	G37.C07	-
Hp_006440225	GCF_006440225_1	Asia	Cambodia	G2	G2.C02	-
Hp_003059525	GCF_003059525_1	Europe	France	G28	G28.C01	-
Hp_006439785	GCF_006439785_1	Asia	Cambodia	G2	G2.C12	-
Hp_002532995	GCF_002532995_1	South America	Colombia	G26	G26.C02	3081
Hp_006438455	GCF_006438455_1	Asia	Cambodia	G2	G2.C03	-
Hp_002357455	GCF_002357455_1	Asia	Japan	G2	G2.C34	-
Hp_002832435	GCF_002832435_1	Asia	India	G3	G3.C12	-
Hp_001446395	GCF_001446395_2	Asia	Malaysia	G2	G2.C14	-
Hp_002357515	GCF_002357515_1	Asia	Japan	G2	G2.C34	-
Hp_000401295	GCF_000401295_1	Asia	India	G3	NA	-
Hp_001940085	GCF_001940085_1	Asia	Singapore	G2	G2.C46	-
Hp_000982695	GCF_000982695_1	North America	United States of America	G37	G37.C25	3496
Hp_004013435	GCF_004013435_1	Europe	Switzerland	G6	NA	-
Hp_006438515	GCF_006438515_1	Asia	Cambodia	G24	G24.C01	-
Hp_009689805	GCF_009689805_1	Asia	China	G2	G2.C36	-
Hp_003639905	GCF_003639905_1	Europe	Spain	G35	G35.C04	-
Hp_000444305	GCF_000444305_1	Asia	Malaysia	G2	G2.C41	3688
Hp_000274965	GCF_000274965_2	North America	United States of America	G37	G37.C31	-
Hp_001653395	GCF_001653395_1	Asia	North Korea	G2	G2.C17	45
Hp_003059335	GCF_003059335_1	Europe	France	NA	NA	-
Hp_001051495	GCF_001051495_1	Asia	Malaysia	G11	G11.C02	-
Hp_002533775	GCF_002533775_1	South America	Colombia	G35	G35.C07	3100
Hp_004012915	GCF_004012915_1	Europe	Switzerland	G7	G7.C01	-

No._Of_Strains	Accession number	Continent	Country	Ggroup	Cgroup	MLST types
Hp_003636925	GCF_003636925_1	Oceania	Australia	G37	G37.C16	-
Hp_004013765	GCF_004013765_1	Europe	Switzerland	G27	G27.C02	-
Hp_006440195	GCF_006440195_1	Asia	Cambodia	G2	G2.C08	-
Hp_006439735	GCF_006439735_1	Asia	Cambodia	G3	NA	-
Hp_002832345	GCF_002832345_1	Asia	India	G11	NA	-
Hp_006439915	GCF_006439915_1	Asia	Cambodia	G3	G3.C02	-
Hp_002832485	GCF_002832485_1	Asia	India	G3	NA	-
Hp_003638115	GCF_003638115_1	Oceania	Australia	G16	G16.C03	-
Hp_002026785	GCF_002026785_1	North America	Mexico	G30	G30.C01	3543
Hp_003639495	GCF_003639495_1	Europe	Spain	G23	G23.C02	-
Hp_006440045	GCF_006440045_1	Asia	Cambodia	G4	G4.C02	-
Hp_004012465	GCF_004012465_1	Europe	Switzerland	G25	G25.C10	-
Hp_003635585	GCF_003635585_1	Oceania	Australia	G8	G8.C02	625
Hp_009689635	GCF_009689635_1	Asia	China	G2	G2.C41	-
Hp_003059265	GCF_003059265_1	Europe	France	NA	0.C10	-
Hp_000689115	GCF_000689115_1	North America	United States of America	G5	G5.C02	-
Hp_004013925	GCF_004013925_1	Europe	Switzerland	G37	G37.C21	-
Hp_003815525	GCF_003815525_1	South America	Colombia	G35	G35.C07	3054
Hp_006337265	GCF_006337265_1	Europe	Germany	G20	G20.C02	-
Hp_002831985	GCF_002831985_1	Asia	India	G11	NA	-
Hp_004012355	GCF_004012355_1	Europe	Switzerland	G23	G23.C01	-
Hp_003636765	GCF_003636765_1	Oceania	Australia	NA	NA	-
Hp_004014025	GCF_004014025_1	Europe	Switzerland	G30	NA	-
Hp_002026825	GCF_002026825_1	North America	Mexico	G36	G36.C02	3551
Hp_002952575	GCF_002952575_1	North America	United States of America	G22	G22.C04	3020
Hp_003637565	GCF_003637565_1	Oceania	Australia	G16	G16.C05	-
Hp_002533105	GCF_002533105_1	South America	Colombia	G35	G35.C05	3144
Hp_003639215	GCF_003639215_1	Europe	Spain	NA	0.C13	-
Hp_003637635	GCF_003637635_1	Oceania	Australia	G25	NA	-
Hp_003815475	GCF_003815475_1	South America	Colombia	G35	G35.C05	3060
Hp_002832595	GCF_002832595_1	Asia	India	G3	G3.C07	-
Hp_003059405	GCF_003059405_1	Europe	France	G17	G17.C02	-
Hp_002357775	GCF_002357775_1	Asia	Japan	G2	G2.C31	-
Hp_003637045	GCF_003637045_1	Oceania	Australia	G2	G2.C01	-
Hp_006337405	GCF_006337405_1	Europe	Germany	G19	G19.C01	-
Hp_009740625	GCF_009740625_1	North America	United States of America	NA	0.C07	-
Hp_008026815	GCF_008026815_1	North America	Mexico	G37	G37.C01	3521
Hp_003058595	GCF_003058595_1	Europe	Sweden	G22	G22.C01	-
Hp_004013305	GCF_004013305_1	Europe	Switzerland	G13	G13.C02	-
Hp_002533285	GCF_002533285_1	South America	Colombia	NA	0.C20	3130
Hp_002534615	GCF_002534615_1	South America	Colombia	G37	G37.C19	3141
Hp_000296015	GCF_000296015_2	Asia	Malaysia	G3	G3.C08	3670
Hp_011140925	GCF_011140925_1	North America	Mexico	G36	G36.C02	3538
Hp_002533515	GCF_002533515_1	South America	Colombia	G35	G35.C06	3116
Hp_008026375	GCF_008026375_1	North America	Mexico	G31	G31.C05	-
Hp_006440005	GCF_006440005_1	Asia	Cambodia	G4	G4.C02	-
Hp_011141045	GCF_011141045_1	North America	Mexico	NA	0.C07	-
Hp_007794935	GCF_007794935_1	Europe	United Kingdom	G22	G22.C04	3020
Hp_003635975	GCF_003635975_1	Oceania	Australia	G2	G2.C06	-
Hp_002533455	GCF_002533455_1	South America	Colombia	G35	G35.C05	3120
Hp_003636165	GCF_003636165_1	Oceania	Australia	G29	G29.C05	-
Hp_003637875	GCF_003637875_1	Oceania	Australia	G29	G29.C05	-
Hp_002533365	GCF_002533365_1	South America	Colombia	G35	G35.C05	3126
Hp_001248025	GCF_001248025_1	Asia	Malaysia	G3	G3.C08	-
Hp_009740365	GCF_009740365_1	North America	United States of America	G37	G37.C20	-
Hp_009741065	GCF_009741065_1	North America	United States of America	G31	G31.C03	-
Hp_000824865	GCF_000824865_1	Europe	United Kingdom	G19	G19.C02	594
Hp_004014275	GCF_004014275_1	Europe	Switzerland	G10	G10.C02	-
Hp_011140485	GCF_011140485_1	North America	Mexico	G36	G36.C03	-
Hp_004013595	GCF_004013595_1	Europe	Switzerland	G7	G7.C03	-
Hp_009690375	GCF_009690375_1	Asia	China	G2	NA	-
Hp_004013075	GCF_004013075_1	Europe	Switzerland	G9	G9.C05	-
Hp_003637435	GCF_003637435_1	Oceania	Australia	G2	G2.C19	-
Hp_003058845	GCF_003058845_1	Europe	Belgium	G15	G15.C01	-
Hp_003059125	GCF_003059125_1	Europe	United Kingdom	G16	G16.C04	-
Hp_011140805	GCF_011140805_1	North America	Mexico	G34	NA	-
Hp_003638235	GCF_003638235_1	Oceania	Australia	G16	G16.C02	1450
Hp_002357395	GCF_002357395_1	Asia	Japan	G2	NA	-
Hp_009741175	GCF_009741175_1	North America	United States of America	G2	G2.C49	-
Hp_003382515	GCF_003382515_1	Europe	Spain	G23	G23.C04	-
Hp_003638175	GCF_003638175_1	Oceania	Australia	G37	G37.C18	-
Hp_003059605	GCF_003059605_1	Europe	France	NA	0.C10	-
Hp_004012855	GCF_004012855_1	Europe	Switzerland	G23	G23.C02	-
Hp_002533425	GCF_002533425_1	South America	Colombia	G35	G35.C07	3122
Hp_000444195	GCF_000444195_2	Asia	China	G2	G2.C42	2566
Hp_000392515	GCF_000392515_3	Asia	Malaysia	G11	G11.C05	-
Hp_002532725	GCF_002532725_1	South America	Colombia	G35	G35.C06	3101
Hp_008681245	GCF_008681245_1	North America	Mexico	G31	NA	-
Hp_000315955	GCF_000315955_1	North America	Canada	G1	G1.C02	2935
Hp_000392455	GCF_000392455_3	Asia	Malaysia	G2	NA	-
Hp_002533655	GCF_002533655_1	South America	Colombia	G35	G35.C07	3110
Hp_001278525	GCF_001278525_1	Asia	Kuwait	G24	G24.C05	-
Hp_009740655	GCF_009740655_1	North America	United States of America	G31	G31.C04	-
Hp_000448425	GCF_000448425_1	South America	Colombia	G37	G37.C23	-
Hp_010917675	GCF_010917675_1	South America	Colombia	G37	G37.C04	-
Hp_002534555	GCF_002534555_1	South America	Colombia	G35	G35.C05	-
Hp_002533985	GCF_002533985_1	South America	Colombia	G35	G35.C05	3078
Hp_000688915	GCF_000688915_1	North America	United States of America	G5	G5.C02	-
Hp_001653455	GCF_001653455_1	South America	Angola	G28	NA	1909
Hp_002012575	GCF_002012575_1	South America	Colombia	G37	G37.C08	3134
Hp_004013845	GCF_004013845_1	Europe	Switzerland	G9	G9.C05	-
Hp_008681355	GCF_008681355_1	North America	Mexico	G33	G33.C02	-
Hp_009740985	GCF_009740985_1	North America	United States of America	G37	G37.C28	-
Hp_011175275	GCF_011175275_1	North America	Nicaragua	G37	G37.C17	-
Hp_004014495	GCF_004014495_1	Europe	Switzerland	NA	NA	-
Hp_002832075	GCF_002832075_1	Asia	India	G3	G3.C11	-
Hp_003636845	GCF_003636845_1	Oceania	Australia	G15	G15.C02	-
Hp_011140955	GCF_011140955_1	North America	Mexico	G25	NA	-
Hp_003639735	GCF_003639735_1	Europe	Spain	G31	G31.C02	-
Hp_009689815	GCF_009689815_1	Asia	China	G2	G2.C37	-
Hp_003639915	GCF_003639915_1	Europe	Spain	G35	NA	-
Hp_011175135	GCF_011175135_1	North America	Nicaragua	G37	G37.C10	-

Hp_000802625	GCF_000802625_1	Europe	Portugal	G27	G27.C02	-
Hp_004012735	GCF_004012735_1	Europe	Switzerland	G25	G25.C10	-
Hp_000148875	GCF_000148875_1	South America	Peru	G1	G1.C01	3041
Hp_006438625	GCF_006438625_1	Asia	Cambodia	G3	G3.C01	-
Hp_009690225	GCF_009690225_1	Asia	China	G2	G2.C07	-
Hp_004014185	GCF_004014185_1	Europe	Switzerland	NA	0.C07	-
Hp_002952395	GCF_002952395_1	North America	United States of America	G22	G22.C03	3020
Hp_002532615	GCF_002532615_1	South America	Colombia	G26	G26.C04	3129
Hp_002533895	GCF_002533895_1	South America	Colombia	G35	G35.C07	3093
Hp_011140435	GCF_011140435_1	North America	Mexico	G37	NA	-
Hp_003635905	GCF_003635905_1	Oceania	Australia	NA	0.C10	-
Hp_003382545	GCF_003382545_1	Europe	Spain	G23	G23.C04	-
Hp_000591335	GCF_000591335_1	Asia	China	G2	G2.C42	2565
Hp_004323355	GCF_004323355_1	Asia	Malaysia	G2	G2.C38	-
Hp_003637805	GCF_003637805_1	Oceania	Australia	G9	NA	-
Hp_000287835	GCF_000287835_1	Asia	China	G2	G2.C19	3673
Hp_004014205	GCF_004014205_1	Europe	Switzerland	G10	G10.C03	-
Hp_000817025	GCF_000817025_1	Asia	China(Taiwan)	G2	NA	-
Hp_002534385	GCF_002534385_1	South America	Colombia	G37	G37.C02	3090
Hp_002534445	GCF_002534445_1	South America	Colombia	G35	G35.C07	3079
Hp_003639325	GCF_003639325_1	Europe	Spain	G32	G32.C02	-
Hp_008681215	GCF_008681215_1	North America	Mexico	G36	G36.C04	-
Hp_004012135	GCF_004012135_1	Europe	Switzerland	NA	0.C11	-
Hp_004012695	GCF_004012695_1	Europe	Switzerland	G21	G21.C01	-
Hp_002533235	GCF_002533235_1	South America	Colombia	G37	G37.C08	3134
Hp_003637225	GCF_003637225_1	Oceania	Australia	G37	G37.C18	-
Hp_010917155	GCF_010917155_1	South America	Colombia	G37	G37.C08	-
Hp_002533175	GCF_002533175_1	South America	Colombia	G35	G35.C05	3136
Hp_003638425	GCF_003638425_1	Oceania	Australia	G12	G12.C02	-
Hp_001189755	GCF_001189755_1	Asia	Malaysia	G2	G2.C38	-
Hp_003637685	GCF_003637685_1	Oceania	Australia	G29	G29.C05	-
Hp_009690075	GCF_009690075_1	Asia	China	G2	G2.C20	-
Hp_003639665	GCF_003639665_1	Europe	Spain	G37	G37.C20	-
Hp_003635855	GCF_003635855_1	Oceania	Australia	G16	G16.C02	-
Hp_000444345	GCF_000444345_1	Asia	Malaysia	G2	G2.C27	3680
Hp_001433495	GCF_001433495_1	North America	Mexico	G27	G27.C02	-
Hp_002534535	GCF_002534535_1	South America	Colombia	G26	G26.C03	-
Hp_003636395	GCF_003636395_1	Oceania	Australia	G29	G29.C04	-
Hp_002026835	GCF_002026835_1	North America	Mexico	G36	G36.C02	3519
Hp_002832505	GCF_002832505_1	Asia	India	G7	G7.C04	-
Hp_001653435	GCF_001653435_1	Oceania	Australia	NA	NA	1047
Hp_003637625	GCF_003637625_1	Oceania	Australia	G28	NA	-
Hp_002012795	GCF_002012795_1	South America	Colombia	G35	G35.C05	3120
Hp_008681335	GCF_008681335_1	North America	Mexico	G35	G35.C06	-
Hp_003637105	GCF_003637105_1	Oceania	Australia	G24	G24.C07	-
Hp_009740565	GCF_009740565_1	North America	United States of America	G37	G37.C29	-
Hp_002533195	GCF_002533195_1	South America	Colombia	NA	0.C07	3135
Hp_000740115	GCF_000740115_1	Asia	China	G2	G2.C04	-
Hp_000451005	GCF_000451005_1	Asia	Malaysia	G11	G11.C05	-
Hp_011140715	GCF_011140715_1	North America	Mexico	G31	G31.C01	-
Hp_003637515	GCF_003637515_1	Oceania	Australia	G2	G2.C08	-
Hp_009690145	GCF_009690145_1	Asia	China	G2	G2.C21	-
Hp_001538305	GCF_001538305_2	Asia	Malaysia	G2	G2.C18	-
Hp_008728675	GCF_008728675_1	North America	Mexico	G30	G30.C01	-
Hp_000591215	GCF_000591215_1	Asia	China	G2	G2.C43	2563
Hp_008027235	GCF_008027235_1	North America	Mexico	G34	G34.C01	-
Hp_001446435	GCF_001446435_2	Asia	Malaysia	G2	G2.C18	-
Hp_003639265	GCF_003639265_1	Europe	Spain	G25	G25.C06	-
Hp_004013395	GCF_004013395_1	Europe	Switzerland	G27	G27.C01	-
Hp_003637285	GCF_003637285_1	Oceania	Australia	G20	G20.C05	-
Hp_002832115	GCF_002832115_1	Asia	India	G3	G3.C12	-
Hp_003638485	GCF_003638485_1	Oceania	Australia	G24	G24.C06	-
Hp_003637745	GCF_003637745_1	Oceania	Australia	G20	G20.C07	-
Hp_003638345	GCF_003638345_1	Oceania	Australia	G37	G37.C28	-
Hp_011140935	GCF_011140935_1	North America	Mexico	G36	G36.C04	-
Hp_011140545	GCF_011140545_1	North America	Mexico	G36	G36.C03	-
Hp_008681525	GCF_008681525_1	North America	Mexico	G30	G30.C01	-
Hp_000281675	GCF_000281675_2	Asia	Malaysia	G3	NA	-
Hp_006440145	GCF_006440145_1	Asia	Cambodia	NA	0.C01	-
Hp_002026675	GCF_002026675_1	North America	Mexico	G31	G31.C01	3536
Hp_002952375	GCF_002952375_1	North America	United States of America	G22	G22.C04	3020
Hp_004117955	GCF_004117955_1	Asia	China(Taiwan)	G2	NA	-
Hp_002534325	GCF_002534325_1	South America	Colombia	G26	G26.C04	3111
Hp_003637365	GCF_003637365_1	Oceania	Australia	G19	G19.C03	-
Hp_004012835	GCF_004012835_1	Europe	Switzerland	G27	G27.C02	-
Hp_003639445	GCF_003639445_1	Europe	Spain	NA	0.C13	-
Hp_006440095	GCF_006440095_1	Asia	Cambodia	G3	G3.C01	-
Hp_003639385	GCF_003639385_1	Europe	Spain	G25	G25.C08	-
Hp_002832395	GCF_002832395_1	Asia	India	G3	G3.C13	-
Hp_009689705	GCF_009689705_1	Asia	China	G2	G2.C09	-
Hp_003636505	GCF_003636505_1	Oceania	Australia	G2	NA	-
Hp_004013115	GCF_004013115_1	Europe	Switzerland	G2	G2.C34	-
Hp_001248035	GCF_001248035_1	Asia	Malaysia	G3	G3.C05	-
Hp_000259235	GCF_000259235_1	Europe	Spain	G26	G26.C02	3021
Hp_003855475	GCF_003855475_1	Asia	China	G2	G2.C28	-
Hp_000689015	GCF_000689015_1	North America	United States of America	G5	G5.C02	204
Hp_005796115	GCF_005796115_2	Europe	Poland	G17	G17.C01	3655
Hp_004013505	GCF_004013505_1	Europe	Switzerland	G13	G13.C02	-
Hp_008033015	GCF_008033015_1	North America	Mexico	G30	G30.C01	-
Hp_009740315	GCF_009740315_1	North America	United States of America	G37	G37.C28	-
Hp_004014605	GCF_004014605_1	Europe	Switzerland	G12	NA	-
Hp_001446375	GCF_001446375_2	Asia	Malaysia	G2	G2.C18	-
Hp_001998045	GCF_001998045_1	South America	Colombia	G37	G37.C15	3508
Hp_008027775	GCF_008027775_1	North America	Mexico	G31	G31.C05	-
Hp_001939635	GCF_001939635_1	Asia	Singapore	G3	G3.C09	-
Hp_002832545	GCF_002832545_1	Asia	India	G8	G8.C01	-
Hp_000256035	GCF_000256035_1	Asia	India	G13	NA	-
Hp_000287735	GCF_000287735_1	Asia	China	G2	G2.C28	2164
Hp_003638305	GCF_003638305_1	Oceania	Australia	NA	NA	-
Hp_001653375	GCF_001653375_1	Asia	India	G2	G2.C01	-
Hp_008681475	GCF_008681475_1	North America	Mexico	G37	G37.C12	-
Hp_000296115	GCF_000296115_2	Asia	Malaysia	G3	G3.C05	-
Hp_001940025	GCF_001940025_1	Asia	Singapore	G2	G2.C47	-
Hp_004012555	GCF_004012555_1	Europe	Switzerland	G14	G14.C01	-
Hp_000148895	GCF_000148895_1	South America	Peru	G1	G1.C02	3040
Hp_000277405	GCF_000277405_1	South America	Peru	G1	G1.C02	3044
Hp_003640045	GCF_003640045_1	Europe	Spain	NA	0.C11	-
Hp_001991095	GCF_001991095_1	Oceania	Australia	G9	G9.C04	-
Hp_004012905	GCF_004012905_1	Europe	Switzerland	G24	G24.C05	-
Hp_002027025	GCF_002027025_1	North America	Mexico	G33	G33.C02	3525
Hp_008032615	GCF_008032615_1	North America	Mexico	NA	0.C14	3527
Hp_003635915	GCF_003635915_1	Oceania	Australia	G37	G37.C24	-
Hp_001189835	GCF_001189835_1	Asia	Malaysia	G3	NA	-
Hp_003059965	GCF_003059965_1	Europe	France	G10	G10.C02	-
Hp_004013225	GCF_004013225_1	Europe	Switzerland	G20	NA	-
Hp_011140845	GCF_011140845_1	North America	Mexico	G31	G31.C05	-
Hp_002532605	GCF_002532605_1	South America	Colombia	G35	G35.C05	3137
Hp_003635735	GCF_003635735_1	Oceania	Australia	G29	G29.C05	-
Hp_003058805	GCF_003058805_1	Europe	United Kingdom	G19	NA	-
Hp_002026665	GCF_002026665_1	North America	Mexico	G33	G33.C02	3535
Hp_004014585	GCF_004014585_1	Europe	Switzerland	G24	G24.C02	-
Hp_003637375	GCF_003637375_1	Oceania	Australia	G37	G37.C33	-
Hp_004012325	GCF_004012325_1	Europe	Switzerland	G23	G23.C03	-
Hp_008032475	GCF_008032475_1	North America	Mexico	G30	G30.C01	-
Hp_002952455	GCF_002952455_1	North America	United States of America	G22	G22.C03	3020
Hp_009740865	GCF_009740865_1	North America	United States of America	G34	NA	-
Hp_002013035	GCF_002013035_1	South America	Colombia	G35	G35.C07	3095
Hp_003636225	GCF_003636225_1	Oceania	Australia	G16	G16.C03	1763
Hp_002012425	GCF_002012425_1	South America	Colombia	G26	G26.C04	-
Hp_004013805	GCF_004013805_1	Europe	Switzerland	G10	G10.C03	-
Hp_001189675	GCF_001189675_1	Asia	Malaysia	G3	G3.C03	-
Hp_002952515	GCF_002952515_1	North America	United States of America	G22	G22.C04	3020
Hp_000287755	GCF_000287755_1	Asia	China	G2	G2.C23	2128
Hp_006440435	GCF_006440435_1	Asia	Cambodia	G2	NA	-
Hp_002357715	GCF_002357715_1	Asia	Japan	G2	G2.C31	-
Hp_006438545	GCF_006438545_1	Asia	Cambodia	G4	G4.C02	-
Hp_008032995	GCF_008032995_1	North America	Mexico	G36	G36.C01	-
Hp_004012535	GCF_004012535_1	Europe	Switzerland	G10	G10.C03	-
Hp_002012655	GCF_002012655_1	South America	Colombia	G35	G35.C07	-
Hp_003058685	GCF_003058685_1	Europe	Sweden	G20	G20.C06	-
Hp_004012665	GCF_004012665_1	Europe	Switzerland	G10	G10.C02	-
Hp_000277385	GCF_000277385_1	South America	Peru	G1	G1.C02	3045
Hp_002534295	GCF_002534295_1	South America	Colombia	G37	G37.C09	3056
Hp_010917555	GCF_010917555_1	South America	Colombia	G37	G37.C09	-
Hp_003636805	GCF_003636805_1	Oceania	Australia	G2	G2.C44	-
Hp_009740575	GCF_009740575_1	North America	United States of America	G37	NA	-
Hp_002533575	GCF_002533575_1	South America	Colombia	G34	G34.C04	3113
Hp_006338945	GCF_006338945_1	Europe	Germany	G20	G20.C02	-
Hp_003637115	GCF_003637115_1	Oceania	Australia	G29	G29.C02	-
Hp_002832285	GCF_002832285_1	Asia	India	G3	G3.C06	-
Hp_001189695	GCF_001189695_1	Asia	Malaysia	G2	NA	-
Hp_002026445	GCF_002026445_1	North America	Mexico	G36	G36.C01	3518
Hp_011175085	GCF_011175085_1	North America	Nicaragua	G27	G27.C02	-
Hp_004014335	GCF_004014335_1	Europe	Switzerland	NA	NA	-
Hp_003058625	GCF_003058625_1	Europe	Sweden	G20	G20.C01	-
Hp_004013365	GCF_004013365_1	Europe	Switzerland	G3	G3.C14	-
Hp_009690065	GCF_009690065_1	Asia	China	G2	G2.C20	-
Hp_003639675	GCF_003639675_1	Europe	Spain	G26	G26.C03	-
Hp_008032975	GCF_008032975_1	North America	Mexico	G35	G35.C03	-
Hp_006337465	GCF_006337465_1	Europe	Germany	G14	NA	-
Hp_002534135	GCF_002534135_1	South America	Colombia	G35	G35.C07	3071
Hp_004012755	GCF_004012755_1	Europe	Switzerland	G25	G25.C05	-
Hp_000296095	GCF_000296095_2	Asia	Malaysia	G3	NA	-
Hp_003637505	GCF_003637505_1	Oceania	Australia	G20	G20.C07	-
Hp_004012265	GCF_004012265_1	Europe	Switzerland	G10	G10.C02	-
Hp_003058745	GCF_003058745_1	Europe	Sweden	G22	G22.C01	-
Hp_003636455	GCF_003636455_1	Oceania	Australia	G2	G2.C35	-
Hp_002533595	GCF_002533595_1	South America	Colombia	G37	G37.C03	3512
Hp_002831955	GCF_002831955_1	Asia	India	G5	NA	-
Hp_001542485	GCF_001542485_2	Asia	Malaysia	G3	G3.C14	-
Hp_004012445	GCF_004012445_1	Europe	Switzerland	G25	G25.C05	-
Hp_008026175	GCF_008026175_1	North America	Mexico	G35	NA	-
Hp_011141115	GCF_011141115_1	North America	Mexico	G37	G37.C07	3537
Hp_003636745	GCF_003636745_1	Oceania	Australia	G2	G2.C09	-
Hp_004013625	GCF_004013625_1	Europe	Switzerland	G17	G17.C02	-
Hp_003059245	GCF_003059245_1	Europe	France	G29	G29.C01	-
Hp_003636285	GCF_003636285_1	Oceania	Australia	G16	G16.C03	1055
Hp_003635555	GCF_003635555_1	Oceania	Australia	G20	G20.C04	1733
Hp_002012485	GCF_002012485_1	South America	Colombia	G35	G35.C07	3110
Hp_004323475	GCF_004323475_1	Asia	Malaysia	G25	G25.C07	-
Hp_004013745	GCF_004013745_1	Europe	Switzerland	G24	G24.C05	-
Hp_002357435	GCF_002357435_1	Asia	Japan	G2	G2.C34	-
Hp_001185025	GCF_001185025_1	Asia	Malaysia	G2	G2.C47	3684
Hp_002026755	GCF_002026755_1	North America	Mexico	G35	G35.C01	3541
Hp_009740305	GCF_009740305_1	North America	United States of America	G25	NA	-
Hp_003060165	GCF_003060165_1	Europe	France	G25	NA	-
Hp_002203985	GCF_002203985_1	Asia	China	G2	G2.C44	-
Hp_003636105	GCF_003636105_1	Oceania	Australia	G4	G4.C01	-
Hp_004323525	GCF_004323525_1	Oceania	Australia	G29	G29.C05	-
Hp_000740095	GCF_000740095_1	Asia	China	G2	G2.C04	-
Hp_002533765	GCF_002533765_1	South America	Colombia	G35	G35.C07	3099
Hp_004012155	GCF_004012155_1	Europe	Switzerland	G17	G17.C02	-
Hp_011174985	GCF_011174985_1	North America	Nicaragua	G24	G24.C06	-
Hp_002027085	GCF_002027085_1	North America	Mexico	G33	G33.C01	3547
Hp_011140625	GCF_011140625_1	North America	Mexico	G30	G30.C01	-
Hp_006440395	GCF_006440395_1	Asia	Cambodia	G4	G4.C02	-
Hp_003058705	GCF_003058705_1	Europe	Sweden	NA	0.C05	-
Hp_004014475	GCF_004014475_1	Europe	Switzerland	G6	NA	-
Hp_011175295	GCF_011175295_1	North America	Nicaragua	G37	NA	3574
Hp_000448485	GCF_000448485_1	South America	Colombia	G35	G35.C06	-
Hp_011140745	GCF_011140745_1	North America	Mexico	G35	G35.C03	-
Hp_003637935	GCF_003637935_1	Oceania	Australia	G20	G20.C04	-
Hp_900450825	GCF_900450825_1	Europe	Belgium	G25	G25.C06	-
Hp_003637545	GCF_003637545_1	Oceania	Australia	G7	G7.C02	-
Hp_003637715	GCF_003637715_1	Oceania	Australia	G25	G25.C01	-
Hp_002533925	GCF_002533925_1	South America	Colombia	G26	G26.C04	3088
Hp_011140515	GCF_011140515_1	North America	Mexico	NA	0.C14	3527
Hp_002831845	GCF_002831845_1	Asia	India	G3	NA	-
Hp_009740925	GCF_009740925_1	North America	United States of America	G37	G37.C27	-
Hp_001998165	GCF_001998165_1	South America	Colombia	G33	G33.C02	3500

Hp_003058715	GCF_003058715_1	Europe	Sweden	G17	G17.C02	-
Hp_002012635	GCF_002012635_1	South America	Colombia	G35	G35.C05	3136
Hp_009690105	GCF_009690105_1	Asia	China	G2	NA	-
Hp_002012565	GCF_002012565_1	South America	Colombia	G35	G35.C05	3117
Hp_009689745	GCF_009689745_1	Asia	China	G2	G2.C29	-
Hp_003636935	GCF_003636935_1	Oceania	Australia	G28	NA	-
Hp_003636545	GCF_003636545_1	Oceania	Australia	G11	G11.C01	-
Hp_011141095	GCF_011141095_1	North America	Mexico	G37	G37.C01	3524
Hp_003639405	GCF_003639405_1	Europe	Spain	NA	0.C06	-
Hp_003636065	GCF_003636065_1	Oceania	Australia	G2	G2.C11	-
Hp_006440105	GCF_006440105_1	Asia	Cambodia	NA	NA	-
Hp_011141145	GCF_011141145_1	North America	Mexico	G30	G30.C01	3530
Hp_006338415	GCF_006338415_1	Europe	Germany	G19	G19.C01	-
Hp_004013065	GCF_004013065_1	Europe	Switzerland	NA	0.C10	-
Hp_002952435	GCF_002952435_1	North America	United States of America	G22	G22.C03	3020
Hp_002013055	GCF_002013055_1	South America	Colombia	NA	0.C19	3109
Hp_002532975	GCF_002532975_1	South America	Colombia	G35	G35.C06	3106
Hp_004013715	GCF_004013715_1	Europe	Switzerland	G37	G37.C24	-
Hp_011105805	GCF_011105805_1	North America	Mexico	G37	G37.C11	-
Hp_006439965	GCF_006439965_1	Asia	Cambodia	G4	G4.C02	-
Hp_004013545	GCF_004013545_1	Europe	Switzerland	G25	NA	-
Hp_009689625	GCF_009689625_1	Asia	China	G2	G2.C40	-
Hp_001939315	GCF_001939315_1	Asia	Singapore	G2	G2.C29	-
Hp_009741165	GCF_009741165_1	North America	United States of America	G37	G37.C30	-
Hp_002012875	GCF_002012875_1	South America	Colombia	G37	G37.C05	3124
Hp_006439645	GCF_006439645_1	Asia	Cambodia	G2	G2.C12	-
Hp_006440285	GCF_006440285_1	Asia	Cambodia	G2	G2.C03	-
Hp_002026555	GCF_002026555_1	North America	Mexico	G35	G35.C02	-
Hp_003638165	GCF_003638165_1	Oceania	Australia	G20	G20.C05	-
Hp_003638595	GCF_003638595_1	Oceania	Australia	G29	G29.C04	-
Hp_008027395	GCF_008027395_1	North America	Mexico	G34	G34.C01	-
Hp_002533815	GCF_002533815_1	South America	Colombia	G37	G37.C08	3098
Hp_003059025	GCF_003059025_1	Europe	Sweden	G20	G20.C06	-
Hp_009740815	GCF_009740815_1	North America	United States of America	G37	G37.C31	-
Hp_004013175	GCF_004013175_1	Europe	Switzerland	G25	G25.C08	-
Hp_003635965	GCF_003635965_1	Oceania	Australia	G10	G10.C03	-
Hp_003637865	GCF_003637865_1	Oceania	Australia	G37	G37.C18	-
Hp_003635645	GCF_003635645_1	Oceania	Australia	G37	G37.C04	-
Hp_009690425	GCF_009690425_1	Asia	China	G2	NA	-
Hp_000689075	GCF_000689075_1	North America	United States of America	G5	G5.C02	-
Hp_008033075	GCF_008033075_1	North America	Mexico	G33	G33.C02	3535
Hp_009689985	GCF_009689985_1	Asia	China	NA	NA	-
Hp_002533375	GCF_002533375_1	South America	Colombia	G37	G37.C05	3124
Hp_002534185	GCF_002534185_1	South America	Colombia	G35	G35.C07	3068
Hp_003639605	GCF_003639605_1	Europe	Spain	G32	G32.C02	-
Hp_006440425	GCF_006440425_1	Asia	Cambodia	G2	G2.C14	-
Hp_002027125	GCF_002027125_1	North America	Mexico	G31	G31.C05	3548
Hp_003637575	GCF_003637575_1	Oceania	Australia	G2	G2.C20	-
Hp_002952195	GCF_002952195_1	North America	United States of America	G22	G22.C04	-
Hp_009690985	GCF_009690985_1	Asia	China	G2	G2.C19	-
Hp_003640145	GCF_003640145_1	Europe	Spain	G36	G36.C03	-
Hp_011105765	GCF_011105765_1	North America	Mexico	G36	G36.C04	3546
Hp_000591275	GCF_000591275_1	Asia	China	G2	G2.C42	2565
Hp_002952635	GCF_002952635_1	North America	United States of America	G22	G22.C03	3020
Hp_011175035	GCF_011175035_1	North America	Nicaragua	G37	G37.C15	-
Hp_004014385	GCF_004014385_1	Europe	Switzerland	G10	G10.C03	-
Hp_003640095	GCF_003640095_1	Europe	Spain	NA	0.C09	-
Hp_002832175	GCF_002832175_1	Asia	India	G3	G3.C10	-
Hp_002831875	GCF_002831875_1	Asia	India	G11	G11.C03	-
Hp_000591135	GCF_000591135_1	Asia	China	G2	G2.C43	2562
Hp_000521245	GCF_000521245_1	Asia	China	G2	G2.C19	2139
Hp_004012475	GCF_004012475_1	Europe	Switzerland	G25	G25.C05	-
Hp_000224575	GCF_000224575_1	Asia	India	G3	NA	3031
Hp_003638225	GCF_003638225_1	Oceania	Australia	G25	NA	-
Hp_011140895	GCF_011140895_1	North America	Mexico	G35	G35.C01	-
Hp_002154535	GCF_002154535_1	Africa	Morocco	G37	G37.C25	3496
Hp_002013135	GCF_002013135_1	South America	Colombia	G35	G35.C07	-
Hp_003070765	GCF_003070765_1	Europe	France	G27	G27.C01	-
Hp_000444745	GCF_000444745_1	Asia	Malaysia	G2	NA	3685
Hp_002532915	GCF_002532915_1	South America	Colombia	G26	G26.C02	3083
Hp_004323385	GCF_004323385_1	Oceania	Australia	G25	G25.C04	-
Hp_004323445	GCF_004323445_1	Oceania	Australia	G10	G10.C01	-
Hp_002012815	GCF_002012815_1	South America	Colombia	G35	G35.C07	3121
Hp_002532735	GCF_002532735_1	South America	Colombia	G35	G35.C07	3059
Hp_001446315	GCF_001446315_2	Asia	Malaysia	G2	G2.C45	-
Hp_002357595	GCF_002357595_1	Asia	Japan	G2	G2.C33	-
Hp_003635885	GCF_003635885_1	Oceania	Australia	G2	G2.C39	-
Hp_003637985	GCF_003637985_1	Oceania	Australia	G2	G2.C32	-
Hp_000444365	GCF_000444365_1	Asia	Malaysia	G2	G2.C07	3681
Hp_003639965	GCF_003639965_1	Europe	Spain	G24	NA	-
Hp_009689865	GCF_009689865_1	Asia	China	G2	NA	-
Hp_008281125	GCF_008281125_1	North America	Mexico	G36	G36.C04	-
Hp_008681415	GCF_008681415_1	North America	Mexico	G37	G37.C12	-
Hp_003639725	GCF_003639725_1	Europe	Spain	NA	0.C11	-
Hp_002534245	GCF_002534245_1	South America	Colombia	G35	G35.C05	3058
Hp_003640105	GCF_003640105_1	Europe	Spain	G9	G9.C05	-
Hp_011174935	GCF_011174935_1	North America	Nicaragua	G37	G37.C10	-
Hp_002533295	GCF_002533295_1	South America	Colombia	NA	0.C06	3128
Hp_002012555	GCF_002012555_1	South America	Colombia	G34	G34.C04	3113
Hp_001653475	GCF_001653475_1	Oceania	Papua New Guinea	NA	NA	1161
Hp_003638405	GCF_003638405_1	Oceania	Australia	G16	G16.C05	-
Hp_006338045	GCF_006338045_1	Europe	Germany	G25	G25.C08	-
Hp_004012635	GCF_004012635_1	Europe	Switzerland	G15	G15.C02	-
Hp_003058585	GCF_003058585_1	Europe	Sweden	G20	G20.C02	-
Hp_004012565	GCF_004012565_1	Europe	Switzerland	G14	G14.C01	-
Hp_003637205	GCF_003637205_1	Oceania	Australia	G20	G20.C07	-
Hp_000392475	GCF_000392475_3	Asia	Singapore	G2	G2.C26	-
Hp_002533675	GCF_002533675_1	South America	Colombia	G35	G35.C07	3110
Hp_011175015	GCF_011175015_1	North America	Nicaragua	G37	G37.C17	-
Hp_002952615	GCF_002952615_1	North America	United States of America	G22	G22.C04	3020
Hp_001940175	GCF_001940175_1	Asia	Singapore	G2	NA	-
Hp_001549715	GCF_001549715_1	Asia	China(Taiwan)	G2	NA	-
Hp_003711165	GCF_003711165_1	Asia	Japan	G3	NA	-
Hp_002832215	GCF_002832215_1	Asia	India	G3	G3.C10	-
Hp_002832055	GCF_002832055_1	Asia	India	G3	G3.C10	-
Hp_001189735	GCF_001189735_1	Asia	Malaysia	G6	G6.C01	-
Hp_002832145	GCF_002832145_1	Asia	India	G4	G4.C01	-
Hp_003637065	GCF_003637065_1	Oceania	Australia	G8	G8.C01	-
Hp_009741045	GCF_009741045_1	North America	United States of America	G37	G37.C14	-
Hp_000591355	GCF_000591355_1	Asia	China	G2	G2.C42	2566
Hp_002013185	GCF_002013185_1	South America	Colombia	G35	G35.C05	3517
Hp_004323335	GCF_004323335_1	Asia	Malaysia	G3	G3.C04	-
Hp_002357375	GCF_002357375_1	Asia	Japan	G2	G2.C38	-
Hp_009798325	GCF_009798325_1	Africa	Morocco	G37	G37.C25	3496
Hp_003638045	GCF_003638045_1	Oceania	Australia	G29	G29.C02	-
Hp_003636705	GCF_003636705_1	Oceania	Australia	G2	G2.C18	-
Hp_004012405	GCF_004012405_1	Europe	Switzerland	G23	G23.C02	-
Hp_006439995	GCF_006439995_1	Asia	Cambodia	NA	NA	-
Hp_000444175	GCF_000444175_1	Asia	China	G2	G2.C43	2561
Hp_003638195	GCF_003638195_1	Oceania	Australia	G20	G20.C07	-
Hp_011140365	GCF_011140365_1	North America	Mexico	G26	G26.C01	3532
Hp_003638565	GCF_003638565_1	Oceania	Australia	G21	G21.C02	758
Hp_003059185	GCF_003059185_1	Europe	United Kingdom	G37	G37.C22	-
Hp_002013205	GCF_002013205_1	South America	Colombia	NA	0.C20	3130
Hp_003636145	GCF_003636145_1	Oceania	Australia	G2	G2.C09	3259
Hp_002357815	GCF_002357815_1	Asia	Japan	G2	G2.C17	-
Hp_009740485	GCF_009740485_1	North America	United States of America	G37	G37.C16	-
Hp_003636095	GCF_003636095_1	Oceania	Australia	G9	G9.C05	-
Hp_001549855	GCF_001549855_1	Asia	China(Taiwan)	G2	G2.C49	-
Hp_009740805	GCF_009740805_1	North America	United States of America	G37	NA	-
Hp_011140425	GCF_011140425_1	North America	Mexico	G37	G37.C06	-
Hp_003957395	GCF_003957395_1	Asia	South Korea	G2	NA	-
Hp_004323415	GCF_004323415_1	Oceania	Australia	G2	G2.C03	-
Hp_000401355	GCF_000401355_1	Asia	Malaysia	G2	G2.C07	3678
Hp_004014675	GCF_004014675_1	Europe	Switzerland	G9	NA	-
Hp_003058865	GCF_003058865_1	Europe	Belgium	G11	NA	-
Hp_003639335	GCF_003639335_1	Europe	Spain	G25	G25.C05	-
Hp_004014295	GCF_004014295_1	Europe	Switzerland	G16	G16.C05	-
Hp_003059905	GCF_003059905_1	Europe	France	G12	G12.C02	-
Hp_002952595	GCF_002952595_1	North America	United States of America	G22	G22.C03	3020
Hp_008032735	GCF_008032735_1	North America	Mexico	G37	G37.C16	-
Hp_003637175	GCF_003637175_1	Oceania	Australia	G32	G32.C01	-
Hp_004012125	GCF_004012125_1	Europe	Switzerland	G25	NA	-
Hp_011105795	GCF_011105795_1	North America	Mexico	G37	G37.C11	3545
Hp_001940185	GCF_001940185_1	Asia	Singapore	G2	G2.C20	-
Hp_001189585	GCF_001189585_1	Asia	Malaysia	G11	NA	-
Hp_002533225	GCF_002533225_1	South America	Colombia	G35	G35.C07	3133
Hp_011140655	GCF_011140655_1	North America	Mexico	G37	G37.C11	-
Hp_003639615	GCF_003639615_1	Europe	Spain	NA	0.C06	-
Hp_002357795	GCF_002357795_1	Asia	Japan	G2	G2.C39	-
Hp_003637235	GCF_003637235_1	Oceania	Australia	G20	G20.C01	-
Hp_002832575	GCF_002832575_1	Asia	India	G7	G7.C04	-
Hp_004012605	GCF_004012605_1	Europe	Switzerland	G17	G17.C02	-
Hp_001051445	GCF_001051445_1	Asia	Malaysia	G2	G2.C14	-
Hp_002534585	GCF_002534585_1	South America	Colombia	G35	G35.C06	3053
Hp_002026885	GCF_002026885_1	North America	Mexico	G31	G31.C01	3523
Hp_002534055	GCF_002534055_1	South America	Colombia	NA	0.C16	3052
Hp_002534215	GCF_002534215_1	South America	Colombia	G35	G35.C06	3065
Hp_004013865	GCF_004013865_1	Europe	Switzerland	G2	G2.C09	-
Hp_002533685	GCF_002533685_1	South America	Colombia	G35	G35.C07	3108
Hp_000091345	GCF_000091345_1	Europe	France	G9	NA	3022
Hp_003639525	GCF_003639525_1	Europe	Spain	G12	G12.C01	-
Hp_009740685	GCF_009740685_1	North America	United States of America	G37	G37.C30	-
Hp_004013655	GCF_004013655_1	Europe	Switzerland	G23	G23.C02	-
Hp_002357315	GCF_002357315_1	Asia	Japan	G2	G2.C31	-
Hp_002026645	GCF_002026645_1	North America	Mexico	G31	G31.C05	3534
Hp_009740845	GCF_009740845_1	North America	United States of America	G37	G37.C27	-
Hp_002831925	GCF_002831925_1	Asia	India	G15	G15.C02	-
Hp_002533845	GCF_002533845_1	South America	Colombia	G35	G35.C05	3097
Hp_011175335	GCF_011175335_1	North America	Nicaragua	G33	G33.C01	-
Hp_003059535	GCF_003059535_1	Europe	France	G12	NA	-
Hp_003636985	GCF_003636985_1	Oceania	Australia	G29	G29.C05	-
Hp_002534495	GCF_002534495_1	South America	Colombia	G35	G35.C06	3076
Hp_002012805	GCF_002012805_1	South America	Colombia	G26	G26.C04	-
Hp_003635615	GCF_003635615_1	Oceania	Australia	G11	G11.C01	-
Hp_002356595	GCF_002356595_1	North America	United States of America	G2	G2.C33	-
Hp_002532905	GCF_002532905_1	South America	Colombia	G35	G35.C05	3080
Hp_002026725	GCF_002026725_1	North America	Mexico	G36	G36.C02	3538
Hp_900478295	GCF_900478295_1	Oceania	Australia	G22	G22.C02	-
Hp_000295995	GCF_000295995_2	Asia	Malaysia	G2	G2.C09	-
Hp_002012955	GCF_002012955_1	South America	Colombia	G35	G35.C06	3127
Hp_003058985	GCF_003058985_1	Europe	Sweden	NA	0.C04	-
Hp_002012685	GCF_002012685_1	South America	Colombia	G35	G35.C05	3138
Hp_001446305	GCF_001446305_2	Asia	Malaysia	G2	G2.C49	-
Hp_003058655	GCF_003058655_1	Europe	Sweden	NA	0.C04	-
Hp_000688995	GCF_000688995_1	North America	United States of America	G5	G5.C02	-
Hp_009737165	GCF_009737165_1	North America	United States of America	G20	G20.C03	2335
Hp_002952255	GCF_002952255_1	North America	United States of America	G22	G22.C04	3020
Hp_004014415	GCF_004014415_1	Europe	Switzerland	G13	G13.C02	-
Hp_000824925	GCF_000824925_1	Europe	United Kingdom	G19	G19.C02	594
Hp_006337325	GCF_006337325_1	Europe	Germany	G24	G24.C08	-
Hp_000472305	GCF_000472305_1	North America	Mexico	G31	G31.C05	-
Hp_002357635	GCF_002357635_1	Asia	Japan	G2	G2.C34	-
Hp_003059325	GCF_003059325_1	Europe	France	G19	NA	-
Hp_003638375	GCF_003638375_1	Oceania	Australia	G14	G14.C02	-
Hp_003637775	GCF_003637775_1	Oceania	Australia	G29	G29.C02	-
Hp_003639995	GCF_003639995_1	Europe	Spain	G32	G32.C02	-
Hp_006438565	GCF_006438565_1	Asia	Cambodia	G4	G4.C02	-
Hp_002209285	GCF_002209285_1	Europe	Switzerland	G9	G9.C04	-
Hp_009690235	GCF_009690235_1	Asia	China	G2	G2.C16	-
Hp_004014375	GCF_004014375_1	Europe	Switzerland	G9	G9.C03	-
Hp_003637055	GCF_003637055_1	Oceania	Australia	G16	G16.C05	-
Hp_000448445	GCF_000448445_1	South America	Colombia	G35	G35.C06	3511
Hp_003639565	GCF_003639565_1	Europe	Spain	NA	NA	-
Hp_003638415	GCF_003638415_1	Oceania	Australia	G25	G25.C08	-
Hp_003639195	GCF_003639195_1	Europe	Spain	G27	G27.C01	-
Hp_008032935	GCF_008032935_1	North America	Mexico	G24	G24.C06	-
Hp_000766025	GCF_000766025_1	Asia	China(Taiwan)	G2	G2.C15	3677
Hp_003639635	GCF_003639635_1	Europe	Spain	G35	G35.C04	-
Hp_004013825	GCF_004013825_1	Europe	Switzerland	G18	G18.C02	-

Hp_003639165	GCF_003639165_1	Europe	Spain	G37	NA	-
Hp_006438645	GCF_006438645_1	Asia	Cambodia	G4	G4.C02	-
Hp_003637825	GCF_003637825_1	Oceania	Australia	G37	G37.C22	-
Hp_004013215	GCF_004013215_1	Europe	Switzerland	G17	G17.C02	-
Hp_002952335	GCF_002952335_1	North America	United States of America	G22	G22.C04	3020
Hp_002204015	GCF_002204015_1	Asia	China	G2	G2.C40	-
Hp_006439955	GCF_006439955_1	Asia	Cambodia	G2	G2.C03	-
Hp_002013065	GCF_002013065_1	South America	Colombia	G35	G35.C07	3091
Hp_000591395	GCF_000591395_1	Asia	China	G2	G2.C43	2562
Hp_004013055	GCF_004013055_1	Europe	Switzerland	G16	G16.C01	-
Hp_009690355	GCF_009690355_1	Asia	China	G2	G2.C17	-
Hp_000148915	GCF_000148915_1	South America	Peru	G1	G1.C02	3043
Hp_006439685	GCF_006439685_1	Asia	Cambodia	NA	NA	-
Hp_006440245	GCF_006440245_1	Asia	Cambodia	G2	G2.C18	-
Hp_003050665	GCF_003050665_1	Oceania	Australia	G22	G22.C02	-
Hp_004012315	GCF_004012315_1	Europe	Switzerland	G31	NA	-
Hp_003636585	GCF_003636585_1	Oceania	Australia	G24	G24.C01	-
Hp_003059545	GCF_003059545_1	Europe	France	NA	0.C11	-
Hp_000317875	GCF_000317875_1	North America	Canada	G1	G1.C02	2945
Hp_003639305	GCF_003639305_1	Europe	Spain	G26	G26.C03	-
Hp_003636215	GCF_003636215_1	Oceania	Australia	G20	G20.C04	664
Hp_003059065	GCF_003059065_1	Europe	Sweden	G20	NA	1233
Hp_002012415	GCF_002012415_1	South America	Colombia	G26	G26.C04	3092
Hp_003635765	GCF_003635765_1	Oceania	Australia	G2	G2.C10	-
Hp_003059715	GCF_003059715_1	Europe	France	G27	G27.C01	-
Hp_009690185	GCF_009690185_1	Asia	China	G2	G2.C17	-
Hp_001998295	GCF_001998295_1	South America	Colombia	G35	G35.C05	3506
Hp_001278515	GCF_001278515_1	Asia	Kuwait	G25	NA	-
Hp_002093735	GCF_002093735_1	Africa	Morocco	G37	G37.C25	3496
Hp_000256075	GCF_000256075_1	Asia	India	G3	NA	-
Hp_004013495	GCF_004013495_1	Europe	Switzerland	G24	G24.C02	-
Hp_000287775	GCF_000287775_1	Asia	China	G2	G2.C19	3674
Hp_001189655	GCF_001189655_1	Asia	Malaysia	G25	G25.C07	-
Hp_009690205	GCF_009690205_1	Asia	China	G2	G2.C20	-
Hp_000296055	GCF_000296055_2	Asia	Malaysia	G2	G2.C21	3671
Hp_000470355	GCF_000470355_1	Europe	Germany	G14	G14.C02	-
Hp_006439845	GCF_006439845_1	Asia	Cambodia	G4	G4.C02	-
Hp_004323225	GCF_004323225_1	Asia	Malaysia	G2	G2.C22	-
Hp_000444325	GCF_000444325_1	Asia	Malaysia	G2	G2.C20	3679
Hp_003635595	GCF_003635595_1	Oceania	Australia	G20	G20.C01	1258
Hp_002951975	GCF_002951975_1	North America	United States of America	G22	G22.C04	-
Hp_002533415	GCF_002533415_1	South America	Colombia	G35	G35.C05	3123
Hp_002831995	GCF_002831995_1	Asia	India	G11	G11.C04	-
Hp_004016925	GCF_004016925_1	Europe	Switzerland	G25	NA	-
Hp_001484135	GCF_001484135_2	Asia	Malaysia	G2	G2.C24	-
Hp_003637465	GCF_003637465_1	Oceania	Australia	G7	NA	-
Hp_002013115	GCF_002013115_1	South America	Colombia	G35	G35.C07	3515
Hp_002952475	GCF_002952475_1	North America	United States of America	G22	G22.C03	3020
Hp_004117965	GCF_004117965_1	Asia	China(Taiwan)	NA	NA	-
Hp_004014075	GCF_004014075_1	Europe	Switzerland	G10	G10.C03	-
Hp_003639485	GCF_003639485_1	Europe	Spain	NA	NA	-
Hp_003059505	GCF_003059505_1	Europe	France	G37	NA	2861
Hp_004013975	GCF_004013975_1	Europe	Switzerland	NA	0.C12	-
Hp_003815575	GCF_003815575_1	South America	Colombia	NA	0.C16	3052
Hp_002532715	GCF_002532715_1	South America	Colombia	G35	G35.C07	3095
Hp_003060085	GCF_003060085_1	Europe	France	G20	G20.C01	-
Hp_004013275	GCF_004013275_1	Europe	Switzerland	G17	G17.C02	-
Hp_000444785	GCF_000444785_1	Asia	Malaysia	G3	G3.C14	3683
Hp_004014595	GCF_004014595_1	Europe	Switzerland	G37	G37.C21	-
Hp_003639805	GCF_003639805_1	Europe	Spain	G33	NA	-
Hp_004013135	GCF_004013135_1	Europe	Switzerland	G25	G25.C09	-
Hp_001248015	GCF_001248015_1	Asia	Malaysia	G3	G3.C04	-
Hp_006338815	GCF_006338815_1	Europe	Germany	G9	G9.C02	-
Hp_000469985	GCF_000469985_1	Europe	Germany	G14	G14.C02	-
Hp_004012375	GCF_004012375_1	Europe	Switzerland	G25	G25.C05	-
Hp_000448525	GCF_000448525_1	Africa	South Africa	G28	G28.C02	3583
Hp_000689035	GCF_000689035_1	North America	United States of America	G5	G5.C02	204
Hp_005796135	GCF_005796135_1	Europe	France	G17	G17.C01	3655
Hp_001185095	GCF_001185095_1	Asia	Malaysia	G3	G3.C14	-
Hp_002012475	GCF_002012475_1	South America	Colombia	G35	G35.C05	3107
Hp_003636275	GCF_003636275_1	Oceania	Australia	NA	0.C11	-
Hp_002533335	GCF_002533335_1	South America	Colombia	G35	G35.C06	3127
Hp_008033035	GCF_008033035_1	North America	Mexico	G31	NA	-
Hp_002534455	GCF_002534455_1	South America	Colombia	G35	G35.C07	3086
Hp_000688955	GCF_000688955_1	North America	United States of America	G5	G5.C02	-
Hp_001653415	GCF_001653415_1	Africa	South Africa	G37	G37.C18	340
Hp_008681315	GCF_008681315_1	North America	Mexico	G33	G33.C02	-
Hp_002532895	GCF_002532895_1	South America	Colombia	G35	G35.C05	3082
Hp_002534515	GCF_002534515_1	South America	Colombia	G37	G37.C02	3067
Hp_004014305	GCF_004014305_1	Europe	Switzerland	G29	G29.C03	-
Hp_002533615	GCF_002533615_1	South America	Colombia	G26	G26.C04	3112
Hp_000591235	GCF_000591235_1	Asia	China	G2	G2.C42	2566
Hp_002952675	GCF_002952675_1	North America	United States of America	G22	G22.C04	3020
Hp_001940115	GCF_001940115_1	Asia	Singapore	G2	G2.C15	-
Hp_011175075	GCF_011175075_1	North America	Nicaragua	G37	G37.C10	-
Hp_003637665	GCF_003637665_1	Oceania	Australia	G28	NA	-
Hp_003635845	GCF_003635845_1	Oceania	Australia	G15	G15.C02	-
Hp_000499325	GCF_000499325_1	Europe	Russia	G2	G2.C01	-
Hp_002832275	GCF_002832275_1	Asia	India	G3	G3.C11	-
Hp_011140735	GCF_011140735_1	North America	Mexico	G30	G30.C01	3530
Hp_000591175	GCF_000591175_1	Asia	China	G2	G2.C43	2562
Hp_011140915	GCF_011140915_1	North America	Mexico	G37	G37.C11	3528
Hp_002533525	GCF_002533525_1	South America	Colombia	G35	G35.C07	3118
Hp_003639685	GCF_003639685_1	Europe	Spain	G28	G28.C01	-
Hp_002534105	GCF_002534105_1	South America	Colombia	G35	G35.C07	3072
Hp_002832135	GCF_002832135_1	Asia	India	G8	G8.C02	-
Hp_002831835	GCF_002831835_1	Asia	India	G3	G3.C07	-
Hp_009740525	GCF_009740525_1	North America	United States of America	G37	NA	-
Hp_000224535	GCF_000224535_1	South America	Peru	G1	G1.C01	-
Hp_004012435	GCF_004012435_1	Europe	Switzerland	G25	G25.C09	-
Hp_004013025	GCF_004013025_1	Europe	Switzerland	G2	G2.C35	-
Hp_004323305	GCF_004323305_1	Oceania	Australia	G6	NA	-
Hp_003639845	GCF_003639845_1	Europe	Spain	G12	G12.C01	-
Hp_009689945	GCF_009689945_1	Asia	China	G2	G2.C30	-
Hp_901687245	GCF_901687245_1	Oceania	Australia	G9	G9.C04	-
Hp_002534175	GCF_002534175_1	South America	Colombia	G26	G26.C04	3066
Hp_003636825	GCF_003636825_1	Oceania	Australia	G30	NA	-
Hp_000148855	GCF_000148855_1	South America	Peru	NA	NA	3050
Hp_002533755	GCF_002533755_1	South America	Colombia	G35	G35.C07	3103
Hp_003640025	GCF_003640025_1	Europe	Spain	NA	0.C19	-
Hp_009740755	GCF_009740755_1	North America	United States of America	G13	NA	-
Hp_011174945	GCF_011174945_1	North America	Nicaragua	G37	G37.C17	-
Hp_002027045	GCF_002027045_1	North America	Mexico	G30	G30.C01	3550
Hp_006439815	GCF_006439815_1	Asia	Cambodia	G2	G2.C09	-
Hp_002534235	GCF_002534235_1	South America	Colombia	NA	0.C16	3052
Hp_002532625	GCF_002532625_1	South America	Colombia	G35	G35.C07	-
Hp_003059145	GCF_003059145_1	Europe	United Kingdom	G19	NA	-
Hp_001998405	GCF_001998405_1	South America	Colombia	G37	G37.C08	3499
Hp_003058825	GCF_003058825_1	Europe	Sweden	NA	0.C04	-
Hp_009740385	GCF_009740385_1	North America	United States of America	G37	G37.C20	-
Hp_004013205	GCF_004013205_1	Europe	Switzerland	G15	G15.C02	-
Hp_003635795	GCF_003635795_1	Oceania	Australia	G6	G6.C01	-
Hp_011141055	GCF_011141055_1	North America	Mexico	G25	NA	-
Hp_003636205	GCF_003636205_1	Oceania	Australia	G20	G20.C07	1889
Hp_002012405	GCF_002012405_1	South America	Colombia	G37	G37.C03	3512
Hp_002013145	GCF_002013145_1	South America	Colombia	G35	G35.C07	3118
Hp_009741085	GCF_009741085_1	North America	United States of America	G37	NA	-
Hp_002026595	GCF_002026595_1	North America	Mexico	G30	G30.C01	3531
Hp_002357475	GCF_002357475_1	Asia	Japan	G2	G2.C13	-
Hp_003638085	GCF_003638085_1	Oceania	Australia	G29	NA	-
Hp_002532745	GCF_002532745_1	South America	Colombia	G35	G35.C07	3061
Hp_006438475	GCF_006438475_1	Asia	Cambodia	G2	G2.C03	-
Hp_008681225	GCF_008681225_1	North America	Mexico	G37	G37.C13	-
Hp_006337365	GCF_006337365_1	Europe	Germany	G25	G25.C11	-
Hp_003059365	GCF_003059365_1	Europe	France	G25	G25.C03	-
Hp_004012255	GCF_004012255_1	Europe	Switzerland	G7	G7.C03	-
Hp_006438525	GCF_006438525_1	Asia	Cambodia	G5	G5.C01	-
Hp_002012755	GCF_002012755_1	South America	Colombia	G34	G34.C02	3140
Hp_000448405	GCF_000448405_1	South America	Colombia	G37	G37.C23	3510
Hp_003059455	GCF_003059455_1	Europe	France	G7	G7.C02	-
Hp_003636355	GCF_003636355_1	Oceania	Australia	G3	G3.C04	-
Hp_004012765	GCF_004012765_1	Europe	Switzerland	G9	G9.C01	-
Hp_003058785	GCF_003058785_1	Europe	United Kingdom	G9	NA	-
Hp_002026485	GCF_002026485_1	North America	Mexico	G30	G30.C01	3530
Hp_003639795	GCF_003639795_1	Europe	Spain	NA	0.C09	-
Hp_002027005	GCF_002027005_1	North America	Mexico	G36	G36.C04	3546
Hp_002357615	GCF_002357615_1	Asia	Japan	G2	G2.C13	-
Hp_000277425	GCF_000277425_1	South America	Peru	G37	NA	3028
Hp_004014435	GCF_004014435_1	Europe	Switzerland	G29	G29.C03	-
Hp_011175045	GCF_011175045_1	North America	Nicaragua	G24	G24.C03	-
Hp_004013465	GCF_004013465_1	Europe	Switzerland	G25	NA	-
Hp_001189775	GCF_001189775_1	Asia	Malaysia	G3	G3.C04	-
Hp_011175195	GCF_011175195_1	North America	Nicaragua	G33	G33.C01	-
Hp_004323375	GCF_004323375_1	Asia	Malaysia	G2	G2.C47	3684
Hp_002357335	GCF_002357335_1	Asia	Japan	G2	G2.C39	-
Hp_003638295	GCF_003638295_1	Oceania	Australia	G37	G37.C30	1213
Hp_002832355	GCF_002832355_1	Asia	India	G11	NA	-
Hp_006337625	GCF_006337625_1	Europe	Germany	G9	G9.C02	-
Hp_006440055	GCF_006440055_1	Asia	Cambodia	G2	NA	-
Hp_009740405	GCF_009740405_1	North America	United States of America	G24	G24.C03	-
Hp_000513515	GCF_000513515_1	Oceania	Australia	G20	G20.C03	1404
Hp_011140825	GCF_011140825_1	North America	Mexico	NA	0.C18	-
Hp_006440215	GCF_006440215_1	Asia	Cambodia	NA	0.C01	-
Hp_011175315	GCF_011175315_1	North America	Nicaragua	G37	G37.C10	-
Hp_003059105	GCF_003059105_1	Europe	Sweden	G17	G17.C01	-
Hp_001998385	GCF_001998385_1	South America	Colombia	G37	G37.C02	3509
Hp_002026925	GCF_002026925_1	North America	Mexico	G35	G35.C02	3526
Hp_001542385	GCF_001542385_2	Asia	Malaysia	G2	G2.C08	-
Hp_006337745	GCF_006337745_1	Europe	Germany	G18	G18.C01	-
Hp_003636785	GCF_003636785_1	Oceania	Australia	G2	G2.C27	-
Hp_006337285	GCF_006337285_1	Europe	Germany	G9	G9.C02	-
Hp_003059285	GCF_003059285_1	Europe	France	G25	G25.C03	-
Hp_003059985	GCF_003059985_1	Europe	France	G23	G23.C01	-
Hp_000590775	GCF_000590775_1	Africa	South Africa	G28	G28.C02	3582
Hp_002534395	GCF_002534395_1	South America	Colombia	G37	G37.C09	3087
Hp_011058095	GCF_011058095_1	South America	Colombia	G35	G35.C06	-
Hp_003636665	GCF_003636665_1	Oceania	Australia	G2	G2.C19	-
Hp_002832325	GCF_002832325_1	Asia	India	G8	G8.C02	-
Hp_000470135	GCF_000470135_1	Europe	Germany	G18	G18.C01	-
Hp_011141135	GCF_011141135_1	North America	Mexico	G37	G37.C06	-
Hp_006440025	GCF_006440025_1	Asia	Cambodia	G5	NA	-
Hp_002191215	GCF_002191215_1	Oceania	Australia	G20	G20.C03	2335
Hp_002357415	GCF_002357415_1	Asia	Japan	G2	G2.C31	-
Hp_004014195	GCF_004014195_1	Europe	Switzerland	G14	NA	-
Hp_004013945	GCF_004013945_1	Europe	Switzerland	G2	G2.C20	-
Hp_006438415	GCF_006438415_1	Asia	Cambodia	NA	0.C02	-
Hp_004014045	GCF_004014045_1	Europe	Switzerland	G24	G24.C01	-
Hp_004013665	GCF_004013665_1	Europe	Switzerland	NA	NA	-
Hp_002026955	GCF_002026955_1	North America	Mexico	G30	G30.C01	3540
Hp_003637695	GCF_003637695_1	Oceania	Australia	G25	G25.C08	-
Hp_002012725	GCF_002012725_1	South America	Colombia	G35	G35.C05	3097
Hp_009740605	GCF_009740605_1	North America	United States of America	G33	G33.C02	-
Hp_006337425	GCF_006337425_1	Europe	Germany	G9	G9.C02	-
Hp_003637135	GCF_003637135_1	Oceania	Australia	G4	G4.C02	-
Hp_003636325	GCF_003636325_1	Oceania	Australia	G37	G37.C16	-
Hp_002533605	GCF_002533605_1	South America	Colombia	G35	G35.C07	-
Hp_003059425	GCF_003059425_1	Europe	France	NA	0.C11	-
Hp_003638475	GCF_003638475_1	Oceania	Australia	G25	G25.C01	-
Hp_000444495	GCF_000444495_1	Asia	Malaysia	G3	NA	3686
Hp_004012225	GCF_004012225_1	Europe	Switzerland	G17	G17.C02	-
Hp_002357555	GCF_002357555_1	Asia	Japan	G2	G2.C34	-
Hp_002026805	GCF_002026805_1	North America	Mexico	G37	G37.C11	3545
Hp_000277365	GCF_000277365_1	South America	Peru	G1	G1.C02	3046
Hp_002012645	GCF_002012645_1	South America	Colombia	G26	G26.C04	3112
Hp_008326525	GCF_008326525_1	Asia	Japan	G2	G2.C23	-
Hp_004012955	GCF_004012955_1	Europe	Switzerland	G25	NA	-
Hp_006338685	GCF_006338685_1	Europe	Germany	G20	G20.C02	-
Hp_004013325	GCF_004013325_1	Europe	Switzerland	G14	NA	-
Hp_002012965	GCF_002012965_1	South America	Colombia	G35	G35.C07	3093
Hp_000730525	GCF_000730525_1	South America	Colombia	G35	G35.C05	3097

Hp_000262655	GCF_000262655_1	Asia	China	G2	NA	3039
Hp_000966875	GCF_000966875_1	North America	United States of America	G22	G22.C04	3020
Hp_004014255	GCF_004014255_1	Europe	Switzerland	G1	NA	-
Hp_002013175	GCF_002013175_1	South America	Colombia	G35	G35.C07	3122
Hp_002952415	GCF_002952415_1	North America	United States of America	G22	G22.C03	3020
Hp_001446295	GCF_001446295_2	Asia	Malaysia	G2	G2.C14	-
Hp_003815515	GCF_003815515_1	South America	Colombia	G37	G37.C08	2958
Hp_000401395	GCF_000401395_1	Asia	Malaysia	G11	G11.C02	-
Hp_003636025	GCF_003636025_1	Oceania	Australia	NA	0.C03	-
Hp_002357755	GCF_002357755_1	Asia	Japan	G2	G2.C33	-
Hp_011105755	GCF_011105755_1	North America	Mexico	G31	G31.C05	3534
Hp_002952555	GCF_002952555_1	North America	United States of America	G22	G22.C03	3020
Hp_000296135	GCF_000296135_2	Asia	Malaysia	G2	G2.C29	-
Hp_002534145	GCF_002534145_1	South America	Colombia	G35	G35.C07	3069
Hp_002832025	GCF_002832025_1	Asia	India	G11	G11.C04	-
Hp_006440325	GCF_006440325_1	Asia	Cambodia	G4	G4.C02	-
Hp_011175225	GCF_011175225_1	North America	Nicaragua	G33	G33.C01	-
Hp_002534095	GCF_002534095_1	South America	Colombia	G35	G35.C05	3073
Hp_011174975	GCF_011174975_1	North America	Nicaragua	G33	G33.C01	-
Hp_002533995	GCF_002533995_1	South America	Colombia	G35	G35.C07	3079
Hp_002012615	GCF_002012615_1	South America	Colombia	NA	0.C19	3131
Hp_006337875	GCF_006337875_1	Europe	Germany	G23	G23.C04	-
Hp_002357535	GCF_002357535_1	Asia	Japan	G2	G2.C32	-
Hp_011140675	GCF_011140675_1	North America	Mexico	G36	G36.C02	-
Hp_001939585	GCF_001939585_1	Asia	Singapore	G2	G2.C48	-
Hp_004013415	GCF_004013415_1	Europe	Switzerland	G25	G25.C10	-
Hp_006438535	GCF_006438535_1	Asia	Cambodia	G2	G2.C08	-
Hp_003636415	GCF_003636415_1	Oceania	Australia	G24	G24.C07	1380
Hp_009737135	GCF_009737135_1	North America	United States of America	G20	G20.C03	2335
Hp_009740975	GCF_009740975_1	North America	United States of America	G37	G37.C28	-
Hp_006338545	GCF_006338545_1	Europe	Germany	G18	G18.C01	-
Hp_002533975	GCF_002533975_1	South America	Colombia	G35	NA	-
Hp_003059195	GCF_003059195_1	Europe	Belgium	G9	G9.C04	-
Hp_003059565	GCF_003059565_1	Europe	France	G37	G37.C24	-
Hp_003636555	GCF_003636555_1	Oceania	Australia	G2	G2.C03	-
Hp_009689755	GCF_009689755_1	Asia	China	G2	G2.C35	-
Hp_002533495	GCF_002533495_1	South America	Colombia	G35	G35.C05	3117
Hp_003636085	GCF_003636085_1	Oceania	Australia	G2	G2.C10	-
Hp_001549875	GCF_001549875_1	Asia	China(Taiwan)	G2	G2.C38	-
Hp_003637405	GCF_003637405_1	Oceania	Australia	G25	G25.C10	-
Hp_003059045	GCF_003059045_1	Europe	Sweden	G20	NA	-
Hp_009741205	GCF_009741205_1	North America	United States of America	G37	G37.C22	-
Hp_009740495	GCF_009740495_1	North America	United States of America	G37	G37.C29	-
Hp_002831915	GCF_002831915_1	Asia	India	G3	G3.C10	-
Hp_004013555	GCF_004013555_1	Europe	Switzerland	G24	NA	-
Hp_006439655	GCF_006439655_1	Asia	Cambodia	G2	G2.C03	-
Hp_002026545	GCF_002026545_1	North America	Mexico	G37	G37.C11	3529
Hp_002026935	GCF_002026935_1	North America	Mexico	G37	G37.C11	3528
Hp_006440265	GCF_006440265_1	Asia	Cambodia	G2	G2.C40	-
Hp_002532795	GCF_002532795_1	South America	Colombia	NA	0.C16	3062
Hp_000401375	GCF_000401375_1	Asia	Malaysia	G2	G2.C18	-
Hp_002013045	GCF_002013045_1	South America	Colombia	G26	G26.C04	3514
Hp_002832415	GCF_002832415_1	Asia	India	G11	NA	-
Hp_004012115	GCF_004012115_1	Europe	Switzerland	G17	G17.C02	-
Hp_000766035	GCF_000766035_1	Asia	Indonesia	G2	G2.C15	-
Hp_002533055	GCF_002533055_1	South America	Colombia	G35	G35.C05	3139
Hp_003639285	GCF_003639285_1	Europe	Spain	G32	G32.C02	-
Hp_003637755	GCF_003637755_1	Oceania	Australia	G37	G37.C33	-
Hp_003638355	GCF_003638355_1	Oceania	Australia	G24	G24.C03	-
Hp_011140555	GCF_011140555_1	North America	Mexico	G36	G36.C04	-
Hp_003639745	GCF_003639745_1	Europe	Spain	NA	0.C16	-
Hp_001189685	GCF_001189685_1	Asia	Malaysia	G2	G2.C18	-
Hp_002832295	GCF_002832295_1	Asia	India	G8	G8.C01	-
Hp_000591195	GCF_000591195_1	Asia	China	G2	G2.C42	2566
Hp_011175145	GCF_011175145_1	North America	Nicaragua	G37	G37.C17	-
Hp_003638465	GCF_003638465_1	Oceania	Australia	G23	G23.C01	-
Hp_003637265	GCF_003637265_1	Oceania	Australia	NA	NA	-
Hp_002534225	GCF_002534225_1	South America	Colombia	G35	G35.C07	3064
Hp_002952275	GCF_002952275_1	North America	United States of America	G22	G22.C04	3020
Hp_003635705	GCF_003635705_1	Oceania	Australia	G29	G29.C04	-
Hp_004013295	GCF_004013295_1	Europe	Switzerland	G25	NA	-
Hp_003639455	GCF_003639455_1	Europe	Spain	G25	G25.C06	-
Hp_003638245	GCF_003638245_1	Oceania	Australia	G20	G20.C04	1145
Hp_004014575	GCF_004014575_1	Europe	Switzerland	NA	NA	-
Hp_006440155	GCF_006440155_1	Asia	Cambodia	G2	G2.C04	-
Hp_011140415	GCF_011140415_1	North America	Mexico	G31	G31.C01	3523
Hp_003639365	GCF_003639365_1	Europe	Spain	G32	G32.C01	-
Hp_004117945	GCF_004117945_1	Asia	China(Taiwan)	G2	G2.C45	-
Hp_000591315	GCF_000591315_1	Asia	China	G2	G2.C43	2562
Hp_001542495	GCF_001542495_2	Asia	Malaysia	G2	G2.C40	-
Hp_000444725	GCF_000444725_1	Asia	Malaysia	G2	G2.C47	3684
Hp_003636675	GCF_003636675_1	Oceania	Australia	G2	G2.C08	-
Hp_002026505	GCF_002026505_1	North America	Mexico	G37	G37.C11	3522
Hp_009690405	GCF_009690405_1	Asia	China	G2	G2.C21	-
Hp_002357855	GCF_002357855_1	Asia	Japan	G2	G2.C13	-
Hp_002012495	GCF_002012495_1	South America	Colombia	G26	G26.C03	3094
Hp_008681235	GCF_008681235_1	South America	Mexico	G30	G30.C01	-
Hp_006440125	GCF_006440125_1	Asia	Cambodia	G2	G2.C03	-
Hp_011141035	GCF_011141035_1	North America	Mexico	G30	G30.C01	-
Hp_003636995	GCF_003636995_1	Oceania	Australia	NA	0.C11	-
Hp_003639425	GCF_003639425_1	Europe	Spain	NA	0.C09	-
Hp_003060025	GCF_003060025_1	Europe	France	G29	G29.C01	-
Hp_001248105	GCF_001248105_1	Asia	Malaysia	G3	NA	-
Hp_002831975	GCF_002831975_1	Asia	India	G11	NA	-
Hp_004014145	GCF_004014145_1	Europe	Switzerland	G24	G24.C06	-
Hp_001997995	GCF_001997995_1	South America	Colombia	G37	NA	3505
Hp_002952495	GCF_002952495_1	North America	United States of America	G22	G22.C03	3020
Hp_009689605	GCF_009689605_1	Asia	China	G2	G2.C05	-
Hp_003638535	GCF_003638535_1	Oceania	Australia	G9	G9.C05	-
Hp_011140335	GCF_011140335_1	North America	Mexico	G28	G28.C01	-
Hp_004014095	GCF_004014095_1	Europe	Switzerland	G23	G23.C03	-
Hp_000401315	GCF_000401315_1	Asia	Malaysia	G3	NA	-
Hp_004323455	GCF_004323455_1	Oceania	Australia	G37	G37.C16	-
Hp_003815595	GCF_003815595_1	South America	Colombia	G35	G35.C07	3105
Hp_002832475	GCF_002832475_1	Asia	India	G3	G3.C13	-

Hp_006439875	GCF_006439875_1	Asia	Cambodia	G4	G4.C02	-
Hp_002532985	GCF_002532985_1	South America	Colombia	G35	G35.C07	3104
Hp_004323315	GCF_004323315_1	Oceania	Australia	G21	G21.C01	-
Hp_001484185	GCF_001484185_2	Asia	Malaysia	G3	G3.C14	-
Hp_006439795	GCF_006439795_1	Asia	Cambodia	G4	G4.C02	-
Hp_011058105	GCF_011058105_1	South America	Colombia	G26	G26.C04	-
Hp_011175375	GCF_011175375_1	North America	Nicaragua	G33	G33.C01	-
Hp_003636905	GCF_003636905_1	Oceania	Australia	G2	G2.C17	-
Hp_003060035	GCF_003060035_1	Europe	France	G15	G15.C01	-
Hp_002012885	GCF_002012885_1	South America	Colombia	G35	G35.C05	-
Hp_009798365	GCF_009798365_1	Africa	Morocco	G37	G37.C25	3496
Hp_004014005	GCF_004014005_1	Europe	Switzerland	NA	0.C07	-
Hp_002357835	GCF_002357835_1	Asia	Japan	G2	G2.C33	-
Hp_004013905	GCF_004013905_1	Europe	Switzerland	G9	G9.C01	-
Hp_003638525	GCF_003638525_1	Oceania	Australia	G19	G19.C03	975
Hp_009689695	GCF_009689695_1	Asia	China	G2	G2.C44	-
Hp_001051485	GCF_001051485_1	Asia	Malaysia	G3	NA	-
Hp_002534545	GCF_002534545_1	South America	Colombia	G35	G35.C07	3055
Hp_008681485	GCF_008681485_1	North America	Mexico	G37	G37.C12	-
Hp_001999845	GCF_001999845_1	South America	Colombia	G35	G35.C06	3497
Hp_003637025	GCF_003637025_1	Oceania	Australia	G2	G2.C20	-
Hp_009740965	GCF_009740965_1	North America	United States of America	NA	0.C14	-
Hp_001998125	GCF_001998125_1	South America	Colombia	G37	G37.C04	3498
Hp_001976105	GCF_001976105_1	Asia	Malaysia	G3	G3.C14	-
Hp_002534065	GCF_002534065_1	South America	Colombia	NA	0.C16	3075
Hp_009740645	GCF_009740645_1	North America	United States of America	G37	G37.C23	-
Hp_003059865	GCF_003059865_1	Europe	France	G29	G29.C01	-
Hp_009690135	GCF_009690135_1	Asia	China	G2	G2.C08	-
Hp_003640585	GCF_003640585_1	Asia	Vietnam	G2	NA	3170
Hp_002357675	GCF_002357675_1	Asia	Japan	G2	G2.C34	-
Hp_001446385	GCF_001446385_2	Asia	Malaysia	G2	G2.C18	-
Hp_009740725	GCF_009740725_1	North America	United States of America	G37	G37.C26	-
Hp_001940145	GCF_001940145_1	Asia	Singapore	G2	G2.C48	-
Hp_003640055	GCF_003640055_1	Europe	Spain	G27	G27.C01	-
Hp_003058905	GCF_003058905_1	Europe	France	G12	G12.C02	-
Hp_011140665	GCF_011140665_1	North America	Mexico	G31	G31.C05	-
Hp_009690275	GCF_009690275_1	Asia	China	G2	NA	-
Hp_003636855	GCF_003636855_2	Oceania	Australia	G29	NA	-
Hp_011140535	GCF_011140535_1	North America	Mexico	G31	G31.C05	-
Hp_001940095	GCF_001940095_1	Asia	Singapore	G2	G2.C46	-
Hp_004013405	GCF_004013405_1	Europe	Switzerland	G17	G17.C02	-
Hp_004012145	GCF_004012145_1	Europe	Switzerland	G17	G17.C02	-
Hp_001446325	GCF_001446325_2	Asia	Malaysia	G2	G2.C45	-
Hp_009740785	GCF_009740785_1	North America	United States of America	G37	G37.C15	-
Hp_001998205	GCF_001998205_1	South America	Colombia	G37	NA	3502
Hp_000688935	GCF_000688935_1	North America	United States of America	G5	G5.C02	-
Hp_002027065	GCF_002027065_1	North America	Mexico	G37	G37.C11	3544
Hp_006338145	GCF_006338145_1	Europe	Germany	G24	G24.C04	-
Hp_002357695	GCF_002357695_1	Asia	Japan	G2	G2.C16	-
Hp_000448465	GCF_000448465_1	South America	Colombia	G37	G37.C04	-
Hp_004012705	GCF_004012705_1	Europe	Switzerland	G18	G18.C02	-
Hp_008681425	GCF_008681425_1	North America	Mexico	G35	G35.C06	-
Hp_002900175	GCF_002900175_1	Europe	Germany	G9	G9.C04	-
Hp_002831855	GCF_002831855_1	Asia	India	G3	G3.C06	-
Hp_002832155	GCF_002832155_1	Asia	India	G7	NA	-
Hp_003059305	GCF_003059305_1	Europe	France	G37	G37.C32	-
Hp_000591255	GCF_000591255_1	Asia	China	G2	G2.C43	2563
Hp_006337305	GCF_006337305_1	Europe	Germany	G24	G24.C08	-
Hp_011140755	GCF_011140755_1	North America	Mexico	NA	0.C17	-
Hp_009689835	GCF_009689835_1	Asia	China	G2	G2.C37	-
Hp_003639935	GCF_003639935_1	Europe	Spain	G32	G32.C02	-
Hp_003639545	GCF_003639545_1	Europe	Spain	G35	NA	-
Hp_004323235	GCF_004323235_1	Asia	Malaysia	G6	G6.C01	-
Hp_011175115	GCF_011175115_1	North America	Nicaragua	G33	G33.C01	-
Hp_006439775	GCF_006439775_1	Asia	Cambodia	G4	G4.C02	-
Hp_004013155	GCF_004013155_1	Europe	Switzerland	G9	G9.C05	-
Hp_003637415	GCF_003637415_1	Oceania	Australia	G29	G29.C02	-
Hp_011175395	GCF_011175395_1	North America	Nicaragua	G37	NA	-
Hp_003638585	GCF_003638585_1	Oceania	Australia	G20	G20.C04	1143
Hp_003638055	GCF_003638055_1	Oceania	Australia	G6	NA	-
Hp_002026705	GCF_002026705_1	North America	Mexico	G37	G37.C07	3537
Hp_000020245	GCF_000020245_1	South America	Peru	G1	G1.C02	3047
Hp_002532925	GCF_002532925_1	South America	Colombia	G35	G35.C05	3084
Hp_008033055	GCF_008033055_1	North America	Mexico	G37	G37.C07	3537
Hp_002533355	GCF_002533355_1	South America	Colombia	G37	G37.C08	3125
Hp_002534435	GCF_002534435_1	South America	Colombia	G35	G35.C07	3089
Hp_003059165	GCF_003059165_1	Europe	Belgium	NA	NA	-
Hp_002832405	GCF_002832405_1	Asia	India	G7	G7.C02	-
Hp_000689055	GCF_000689055_1	North America	United States of America	G5	G5.C02	204
Hp_002533465	GCF_002533465_1	South America	Colombia	G35	G35.C07	3121
Hp_003636155	GCF_003636155_1	Oceania	Australia	G15	G15.C02	-
Hp_000392535	GCF_000392535_3	Asia	Malaysia	G2	G2.C25	3682
Hp_009740465	GCF_009740465_1	North America	United States of America	G37	G37.C20	-
Hp_006440415	GCF_006440415_1	Asia	Cambodia	G5	G5.C01	-
Hp_001998245	GCF_001998245_1	South America	Colombia	G35	G35.C05	3503
Hp_002952535	GCF_002952535_1	North America	United States of America	G22	G22.C04	3020
Hp_002026455	GCF_002026455_1	North America	Mexico	NA	0.C14	3527
Hp_002533205	GCF_002533205_1	South America	Colombia	G26	G26.C04	3132
Hp_003637295	GCF_003637295_1	Oceania	Australia	G25	NA	-
Hp_002357735	GCF_002357735_1	Asia	Japan	G2	G2.C34	-
Hp_002005525	GCF_002005525_1	Oceania	Australia	G9	G9.C04	-
Hp_006338285	GCF_006338285_1	Europe	Germany	G21	G21.C01	-
Hp_006337345	GCF_006337345_1	Europe	Germany	G9	G9.C02	-
Hp_003059485	GCF_003059485_1	Europe	France	G12	NA	-
Hp_004012515	GCF_004012515_1	Europe	Switzerland	G16	G16.C01	-
Hp_003636445	GCF_003636445_1	Oceania	Australia	G25	G25.C04	3153
Hp_003636385	GCF_003636385_1	Oceania	Australia	G29	G29.C05	-
Hp_006337485	GCF_006337485_1	Europe	Germany	G15	G15.C01	-
Hp_003058755	GCF_003058755_1	Europe	Sweden	NA	0.C05	958
Hp_005250645	GCF_005250645_1	Europe	Belarus	NA	0.C05	-
Hp_006439895	GCF_006439895_1	Asia	Cambodia	G4	G4.C01	-
Hp_001542465	GCF_001542465_2	Asia	Malaysia	G2	G2.C08	-
Hp_002534375	GCF_002534375_1	South America	Colombia	G26	G26.C04	3514
Hp_004012365	GCF_004012365_1	Europe	Switzerland	G25	G25.C05	-
Hp_001420425	GCF_001420425_2	Asia	Malaysia	G2	G2.C45	-

Hp_003637965	GCF_003637965_1	Oceania	Australia	G21	G21.C02	-
Hp_008032955	GCF_008032955_1	North America	Mexico	NA	0.C18	-
Hp_002533025	GCF_002533025_1	South America	Colombia	G35	G35.C07	3105
Hp_004323515	GCF_004323515_1	Oceania	Australia	G10	G10.C01	-
Hp_001189765	GCF_001189765_1	Asia	Malaysia	G2	G2.C22	-
Hp_011140615	GCF_011140615_1	North America	Mexico	G36	G36.C01	-
Hp_000444385	GCF_000444385_1	Asia	Malaysia	G3	G3.C10	-
Hp_003639985	GCF_003639985_1	Europe	Spain	NA	0.C06	-
Hp_009689885	GCF_009689885_1	Asia	China	G2	G2.C30	-
Hp_003058995	GCF_003058995_1	Europe	Sweden	NA	0.C05	-
Hp_009690125	GCF_009690125_1	Asia	China	G2	G2.C09	-
Hp_009740735	GCF_009740735_1	North America	United States of America	G37	G37.C26	-
Hp_004012775	GCF_004012775_1	Europe	Switzerland	G18	G18.C02	-
Hp_002533915	GCF_002533915_1	South America	Colombia	G35	G35.C07	3091
Hp_004012895	GCF_004012895_1	Europe	Switzerland	G9	G9.C03	-
Hp_003636525	GCF_003636525_1	Oceania	Australia	NA	0.C03	-
Hp_006439745	GCF_006439745_1	Asia	Cambodia	G2	G2.C02	-
Hp_002534305	GCF_002534305_1	South America	Colombia	G35	G35.C05	3102
Hp_002357355	GCF_002357355_1	Asia	Japan	G2	G2.C39	-
Hp_006438465	GCF_006438465_1	Asia	Cambodia	G2	G2.C03	-
Hp_011140475	GCF_011140475_1	North America	Mexico	NA	0.C08	-
Hp_004014285	GCF_004014285_1	Europe	Switzerland	G25	NA	-
Hp_004088075	GCF_004088075_1	Asia	China	G2	G2.C19	-
Hp_000591375	GCF_000591375_1	Asia	China	G2	G2.C43	2561
Hp_004014625	GCF_004014625_1	Europe	Switzerland	G10	G10.C03	-
Hp_003637885	GCF_003637885_1	Oceania	Australia	G37	G37.C33	-
Hp_003636615	GCF_003636615_1	Oceania	Australia	G16	G16.C03	3255
Hp_001185015	GCF_001185015_1	Asia	Malaysia	G6	G6.C01	-
Hp_004013525	GCF_004013525_1	Europe	Switzerland	G25	G25.C05	-
Hp_009689965	GCF_009689965_1	Asia	China	G2	G2.C07	-
Hp_003639865	GCF_003639865_1	Europe	Spain	NA	0.C08	-
Hp_004014105	GCF_004014105_1	Europe	Switzerland	G7	G7.C01	-
Hp_003635655	GCF_003635655_1	Oceania	Australia	G13	G13.C02	-
Hp_008681395	GCF_008681395_1	North America	Mexico	G35	G35.C06	-
Hp_000451025	GCF_000451025_1	Asia	Malaysia	G2	G2.C25	3682
Hp_002532815	GCF_002532815_1	South America	Colombia	G35	G35.C05	-
Hp_002533275	GCF_002533275_1	South America	Colombia	NA	0.C19	3131
Hp_002026895	GCF_002026895_1	North America	Mexico	G37	G37.C01	3524
Hp_000333835	GCF_000333835_1	Europe	Russia	NA	NA	1767
Hp_003636335	GCF_003636335_1	Oceania	Australia	G15	G15.C02	-
Hp_002533135	GCF_002533135_1	South America	Colombia	G34	G34.C02	3140
Hp_003637605	GCF_003637605_1	Oceania	Australia	G24	NA	-
Hp_002832525	GCF_002832525_1	Asia	India	G25	G25.C02	-
Hp_002533695	GCF_002533695_1	South America	Colombia	G35	G35.C05	3107
Hp_002012915	GCF_002012915_1	South America	Colombia	G35	G35.C05	3123
Hp_000685665	GCF_000685665_1	Oceania	Australia	G13	G13.C01	-
Hp_009690965	GCF_009690965_1	Asia	Japan	G2	G2.C31	-
Hp_003640155	GCF_003640155_1	Europe	Spain	NA	0.C13	-
Hp_011140995	GCF_011140995_1	North America	Mexico	G34	G34.C03	3520
Hp_002832615	GCF_002832615_1	Asia	India	G8	G8.C02	-
Hp_003639575	GCF_003639575_1	Europe	Spain	NA	NA	-
Hp_003815485	GCF_003815485_1	South America	Colombia	G35	G35.C05	-
Hp_000295975	GCF_000295975_2	Asia	Malaysia	G3	G3.C04	3669
Hp_002533095	GCF_002533095_1	South America	Colombia	G35	G35.C07	3142
Hp_002012745	GCF_002012745_1	South America	Colombia	G35	G35.C05	3139
Hp_003059385	GCF_003059385_1	Europe	France	G13	NA	-
Hp_006337445	GCF_006337445_1	Europe	Germany	G24	G24.C08	-
Hp_006337385	GCF_006337385_1	Europe	Germany	G24	G24.C04	-
Hp_003059445	GCF_003059445_1	Europe	France	G15	G15.C02	-
Hp_001976125	GCF_001976125_1	Asia	Malaysia	G3	G3.C14	-
Hp_003636485	GCF_003636485_1	Oceania	Australia	G20	G20.C01	-
Hp_004012785	GCF_004012785_1	Europe	Switzerland	G9	G9.C03	-
Hp_000439295	GCF_000439295_2	Asia	Singapore	G2	G2.C26	-
Hp_002533145	GCF_002533145_1	South America	Colombia	G35	G35.C05	3138
Hp_006339365	GCF_006339365_1	Europe	Germany	G14	NA	-
Hp_004012245	GCF_004012245_1	Europe	Switzerland	G25	NA	-
Hp_000688975	GCF_000688975_1	North America	United States of America	G5	G5.C02	-
Hp_011175215	GCF_011175215_1	North America	Nicaragua	G37	NA	-
Hp_006440315	GCF_006440315_1	Asia	Cambodia	G2	G2.C03	-
Hp_001998285	GCF_001998285_1	South America	Colombia	G37	G37.C08	3501
Hp_000591155	GCF_000591155_1	Asia	China	G2	G2.C43	2561
Hp_009740705	GCF_009740705_1	North America	United States of America	G37	G37.C30	-
Hp_009689875	GCF_009689875_1	Asia	China	G2	G2.C35	-
Hp_002832255	GCF_002832255_1	Asia	India	G15	G15.C02	-
Hp_001940035	GCF_001940035_1	Asia	Singapore	G3	G3.C09	-
Hp_011140595	GCF_011140595_1	North America	Mexico	NA	0.C15	3549
Hp_004323275	GCF_004323275_1	Asia	Malaysia	G3	G3.C04	-
Hp_009690045	GCF_009690045_1	Asia	China	G2	G2.C21	-
Hp_002952655	GCF_002952655_1	North America	United States of America	G22	G22.C04	3020
Hp_002533705	GCF_002533705_1	South America	Colombia	G26	G26.C03	-
Hp_000255955	GCF_000255955_1	North America	El Salvador	NA	NA	3048
Hp_004013015	GCF_004013015_1	Europe	Switzerland	NA	0.C11	-
Hp_009690315	GCF_009690315_1	Asia	China	G2	G2.C36	-
Hp_003637835	GCF_003637835_1	Oceania	Australia	G29	G29.C02	-
Hp_002026625	GCF_002026625_1	North America	Mexico	G26	G26.C01	3532
Hp_003635545	GCF_003635545_1	Oceania	Australia	G16	G16.C04	1883
Hp_001189815	GCF_001189815_1	Asia	Malaysia	G2	G2.C44	-
Hp_003635715	GCF_003635715_1	Oceania	Australia	G37	G37.C33	-
Hp_000224555	GCF_000224555_1	South America	Peru	G1	G1.C02	3042
Hp_008026615	GCF_008026615_1	North America	Mexico	G37	G37.C11	3522
Hp_003636265	GCF_003636265_1	Oceania	Australia	G16	G16.C03	-
Hp_002533825	GCF_002533825_1	South America	Colombia	G35	G35.C07	3096
Hp_002026515	GCF_002026515_1	North America	Mexico	G37	G37.C01	3521
Hp_003059925	GCF_003059925_1	Europe	France	G12	G12.C02	-
Hp_900638475	GCF_900638475_1	Africa	Nigeria	G29	G29.C01	1288
Hp_002532595	GCF_002532595_1	South America	Colombia	NA	NA	-
Hp_004014175	GCF_004014175_1	Europe	Switzerland	G12	NA	-
Hp_002013015	GCF_002013015_1	South America	Colombia	G35	G35.C07	3096
Hp_004013755	GCF_004013755_1	Europe	Switzerland	NA	NA	-
Hp_000258845	GCF_000258845_1	Oceania	Australia	G11	NA	415
Hp_002026745	GCF_002026745_1	North America	Mexico	G36	G36.C01	3539
Hp_006439715	GCF_006439715_1	Asia	Cambodia	G2	G2.C02	-
Hp_004014545	GCF_004014545_1	Europe	Switzerland	G24	G24.C02	-
Hp_009740855	GCF_009740855_1	North America	United States of America	G31	G31.C03	-
Hp_001997965	GCF_001997965_1	South America	Colombia	G37	G37.C23	3504
Hp_002026985	GCF_002026985_1	North America	Mexico	G37	G37.C13	3542
Hp_002533855	GCF_002533855_1	South America	Colombia	G26	G26.C03	3094
Hp_003636725	GCF_003636725_1	Oceania	Australia	G15	G15.C01	-
Hp_001433515	GCF_001433515_1	North America	Mexico	NA	0.C07	-
Hp_003638135	GCF_003638135_1	Oceania	Australia	G29	G29.C04	-
Hp_002952355	GCF_002952355_1	North America	United States of America	G22	G22.C04	3020
Hp_003059225	GCF_003059225_1	Europe	France	G37	NA	-
Hp_004013965	GCF_004013965_1	Europe	Switzerland	G10	G10.C01	-
Hp_001185045	GCF_001185045_1	Asia	Malaysia	G3	NA	-
Hp_003636645	GCF_003636645_1	Oceania	Australia	G2	G2.C11	-
Hp_003636965	GCF_003636965_1	Oceania	Australia	G37	G37.C18	-
Hp_000689135	GCF_000689135_1	North America	United States of America	G5	G5.C02	-
Hp_003636035	GCF_003636035_1	Oceania	Australia	G6	NA	-
Hp_009689615	GCF_009689615_1	Asia	China	G2	G2.C27	-
Hp_004012985	GCF_004012985_1	Europe	Switzerland	G9	G9.C05	-
Hp_003058965	GCF_003058965_1	Europe	Sweden	NA	0.C04	-
Hp_002012945	GCF_002012945_1	South America	Colombia	G35	G35.C07	3055
Hp_004012655	GCF_004012655_1	Europe	Switzerland	G24	G24.C05	-
Hp_002983705	GCF_002983705_1	North America	United States of America	G37	G37.C25	3496
Hp_003637995	GCF_003637995_1	Oceania	Australia	G2	G2.C08	-
Hp_009740905	GCF_009740905_1	North America	United States of America	G31	G31.C04	-
Hp_002534005	GCF_002534005_1	South America	Colombia	G37	G37.C05	3077
Hp_011175235	GCF_011175235_1	North America	Nicaragua	G34	NA	-
Hp_006440335	GCF_006440335_1	Asia	Cambodia	G2	G2.C03	-
Hp_002533905	GCF_002533905_1	South America	Colombia	G26	G26.C04	3092
Hp_011175175	GCF_011175175_1	North America	Nicaragua	G37	G37.C11	-
Hp_002832645	GCF_002832645_1	Asia	India	G11	NA	-
Hp_001940015	GCF_001940015_1	Asia	Singapore	G2	G2.C15	-
Hp_009741025	GCF_009741025_1	North America	United States of America	G2	G2.C23	-
Hp_002534315	GCF_002534315_1	South America	Colombia	G26	G26.C04	3513
Hp_003638025	GCF_003638025_1	Oceania	Australia	G4	G4.C02	-
Hp_004013605	GCF_004013605_1	Europe	Switzerland	NA	0.C06	-
Hp_008681515	GCF_008681515_1	North America	Mexico	NA	0.C17	-
Hp_002222575	GCF_002222575_1	Asia	China	G2	G2.C05	-
Hp_008681285	GCF_008681285_1	North America	Mexico	G36	G36.C04	-
Hp_009689995	GCF_009689995_1	Asia	China	G2	G2.C30	-
Hp_004014505	GCF_004014505_1	Europe	Switzerland	G25	NA	-
Hp_004012865	GCF_004012865_1	Europe	Switzerland	G10	G10.C01	-
Hp_003058885	GCF_003058885_1	Europe	Belgium	G27	G27.C02	-
Hp_002532685	GCF_002532685_1	South America	Colombia	G35	G35.C07	3057
Hp_005796125	GCF_005796125_1	Europe	Poland	G17	G17.C01	3655
Hp_002012855	GCF_002012855_1	South America	Colombia	G37	G37.C08	3098
Hp_002357495	GCF_002357495_1	Asia	Japan	G2	G2.C16	-
Hp_004014115	GCF_004014115_1	Europe	Switzerland	NA	0.C09	-
Hp_001484155	GCF_001484155_2	Asia	Malaysia	G2	G2.C24	-
Hp_009740325	GCF_009740325_1	North America	United States of America	G37	G37.C14	-
Hp_003636605	GCF_003636605_1	Oceania	Australia	G2	G2.C03	-
Hp_004014395	GCF_004014395_1	Europe	Switzerland	G15	G15.C02	-
Hp_004323545	GCF_004323545_1	Oceania	Australia	G3	NA	-
Hp_003636885	GCF_003636885_1	Oceania	Australia	G21	G21.C02	-
Hp_003059845	GCF_003059845_1	Europe	Ireland	G20	G20.C07	-
Hp_002532805	GCF_002532805_1	South America	Colombia	G35	G35.C05	3070
Hp_003640085	GCF_003640085_1	Europe	Spain	G31	G31.C02	-
Hp_008681365	GCF_008681365_1	North America	Mexico	G33	G33.C02	-
Hp_002832605	GCF_002832605_1	Asia	India	G25	G25.C02	-
Hp_004012575	GCF_004012575_1	Europe	Switzerland	G32	G32.C01	-
Hp_002026865	GCF_002026865_1	North America	Mexico	NA	0.C15	3549
Hp_002533535	GCF_002533535_1	South America	Colombia	NA	0.C20	3115
Hp_001999865	GCF_001999865_1	South America	Colombia	G37	G37.C19	3507
Hp_009740535	GCF_009740535_1	North America	United States of America	G37	G37.C26	-
Hp_000296035	GCF_000296035_2	Asia	Malaysia	G2	G2.C41	-
Hp_008681455	GCF_008681455_1	North America	Mexico	G37	G37.C12	-

Supplementary Table 2. Genomes information for testing

GenBank assembly accession	Genome submit date	Collected continent	Collected country	Sequencing platform
GCF_002206465.1	19/01/2018	North America	United States of America	sequenced by Pacbio & Illumina
GCF_002900195.1	25/01/2018	Europe	Germany	sequenced by Illumina
GCF_004307235.1	31/01/2019	Aisa	Japan	sequenced by Illumina
GCF_004307275.1	31/01/2019	Aisa	Japan	sequenced by Illumina
GCF_004307295.1	31/01/2019	Aisa	Japan	sequenced by Illumina
GCF_900478295.1	18/06/2018	Oceania	Australia	sequenced by Illumina
GCF_016757535.1	27/01/2021	South America	Peru	sequenced by Illumina
GCF_016749335.1	27/01/2021	South America	Peru	sequenced by Illumina
GCF_016757535.1	27/01/2021	South America	Peru	sequenced by Illumina
GCF_013122115.1	19/05/2020	Europe	Russia	sequenced by Illumina

User instruction

Helicobactor pylori typing tool (HPTT) is a genomic typing method based on whole genome sequencing (WGS) data, which can facilitate genetic population analysis and epidemiological origin tracing. In our *H. pylori* typing website (https://db.cngb.org/HPTT/), two modules are available, including both genetic typing and geographic visualizing of the global *H. pylori* isolates. Here is the website instruction for users.

Homepage

You can see the brief introduction of our HPTT on the homepage of our website. Two modules were displayed at this page: the genomic typing tool of *H. pylori* ("QUERY TOOL") and the phylogeographic visualization tool of *H. pylori* ("VISUALIZATION TOOL").

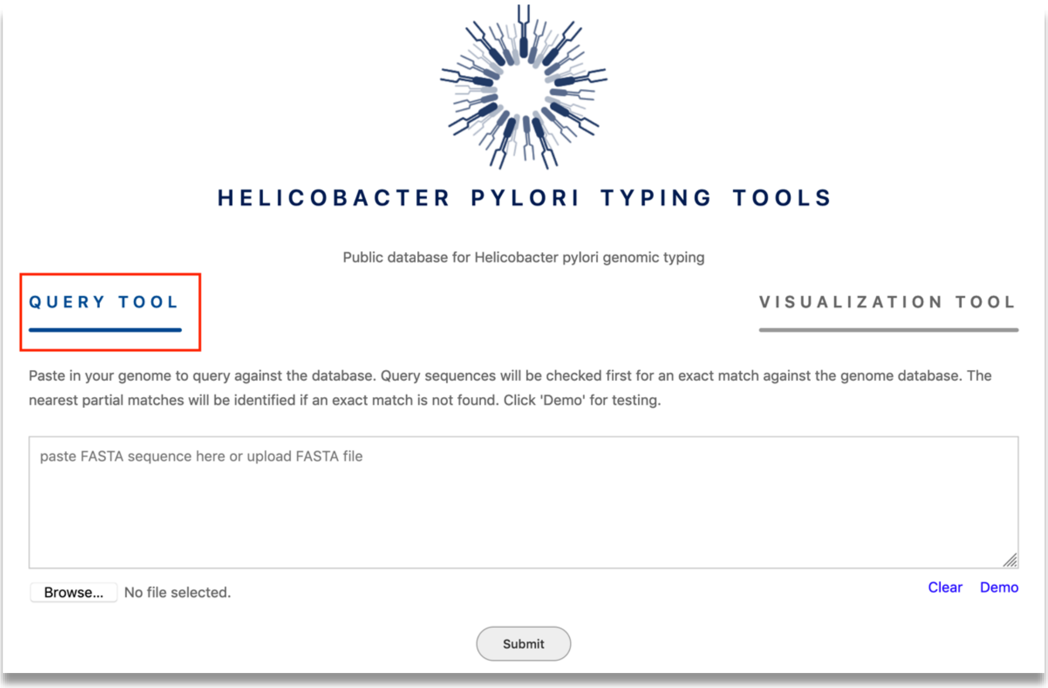

- Data compatibility

In order to achieve the best typing results, it is highly recommended to upload sequencing data generated by the MPS (Massive Parallel Sequencing) method. The common MPS platform includes MGI DNBSEQ® platform and Illumina® platform. General workflow for the MPS including sample pre-treatment, library preparation, sequencing and data analysis. Companies who doing MPS would offer the solutions for all steps, including reagent, instrument, etc.

- The genomic typing tool of *H. pylori*

At the homepage of website, a tested demo genome is provided. Please see the screenshot below. Click the "Demo" button at the bottom right corner of the input box. It may take few seconds since the genome file is relatively large, and there might be a slight freeze during the loading. If the genome is entered incorrectly, click the "Clear" button just next to the “Demo” to clear the input box. For your own sequenced genome, you can either copy and paste in the input box or upload it by click the “Browse” button. After entering/ uploading the genome, click the “Submit” button to query against the database.

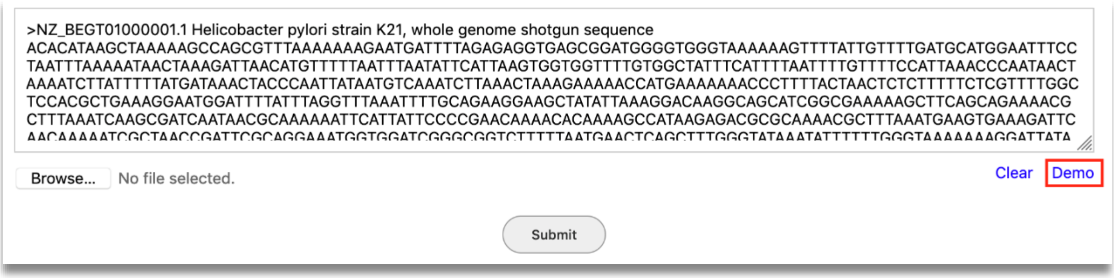

If your uploaded file is neither *H. pylori* nor sequenced by the second-generation sequencing technology, it might be reported as an error. You will need to check whether your genome fit our criteria.

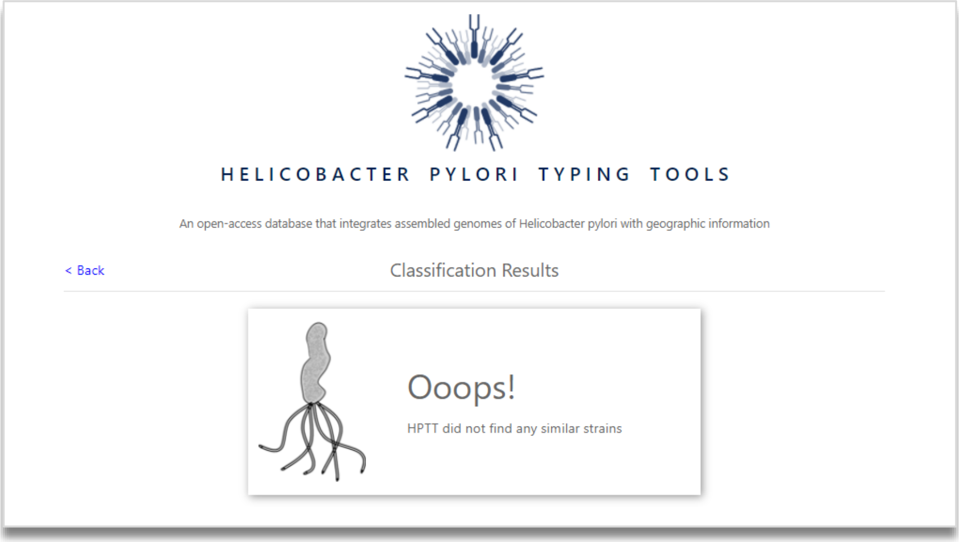

You will get the typing results if it is successfully typed. Please see the image below. Firstly, the strain with the highest identity ("the closest matching strain") that is most similar to our database will be displayed, including region, country, MLST type, data release time, sequenced research institution, etc. Secondly, if the most similar bacterial species has a typing patterns in our database, it will be displayed on the right. If the corresponding types not completely refer to one continent/country of patterns in our database, the proportion of each continent/country will be displayed in different colours in the bar.

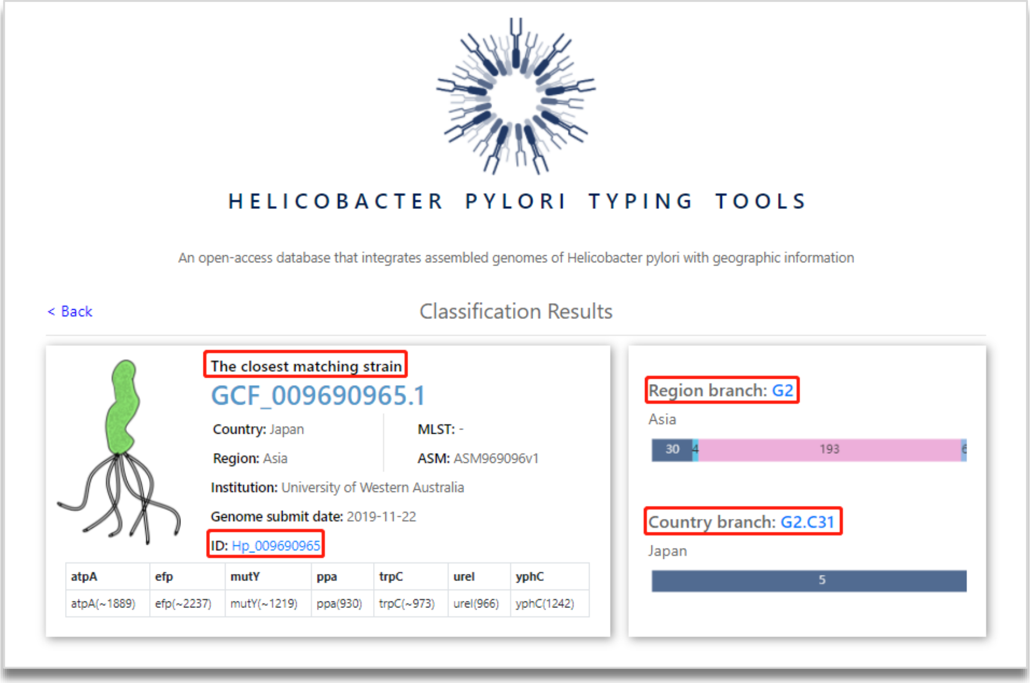

For the region and country groups in our database, each strain can be listed to the corresponding groups. In addition, you can easily access to the visualization tool and NCBI information page by clicking the link (letters in blue).

G2 Region Group List - 233 strains

Group	Genome_id	Tip_id	Region	Country	Submit_date	MLST	Link(NCBI)
G2	GCF_000262655.1	Hp_000262655	Asia	China	2012-05-14	3039	more
G2	GCF_000287735.1	Hp_000287735	Asia	China	2013-02-23	2164	more
G2	GCF_000287755.1	Hp_000287755	Asia	China	2013-02-23	2128	more

G2.C31 Country Group List - 5 strains

Group	Genome_id	Tip_id	Region	Country	Submit_date	MLST	Link(NCBI)
G2.C31	GCF_002357315.1	Hp_002357315	Asia	Japan	2017-01-17	-	more
G2.C31	GCF_002357415.1	Hp_002357415	Asia	Japan	2017-01-17	-	more
G2.C31	GCF_002357715.1	Hp_002357715	Asia	Japan	2017-01-17	-	more
G2.C31	GCF_002357775.1	Hp_002357775	Asia	Japan	2017-01-17	-	more
G2.C31	GCF_009690965.1	Hp_009690965	Asia	Japan	2019-11-22	-	more

- The visualization tool of *H. pylori*

You can easily access to the visualization tool at the homepage by clicking the "VISUALIZATION TOOL" button. All the genomes in our database can be interacted and displayed in both phylogenetic relationships and geographic locations.

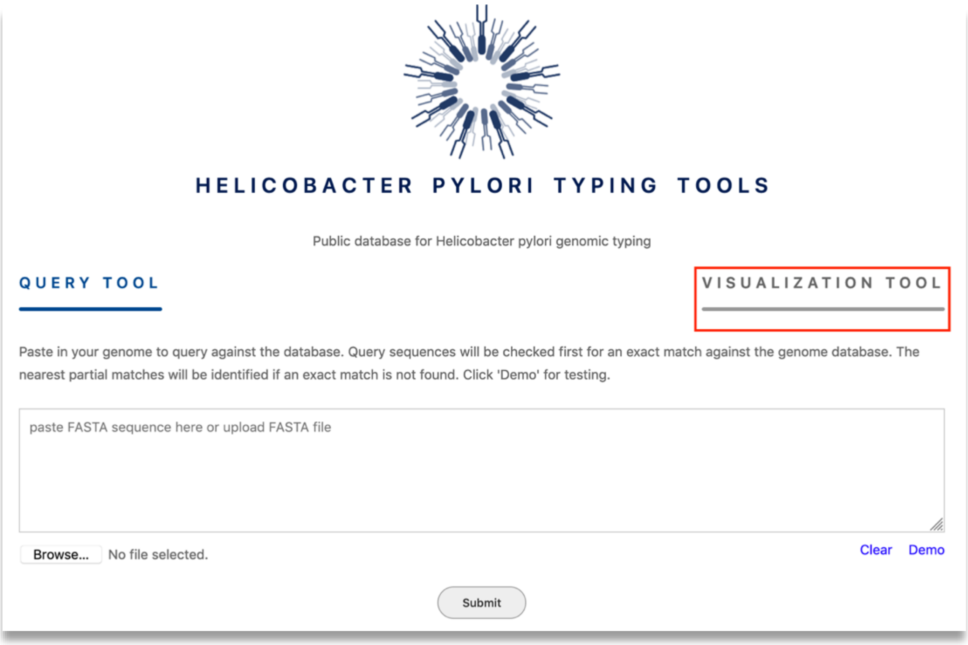

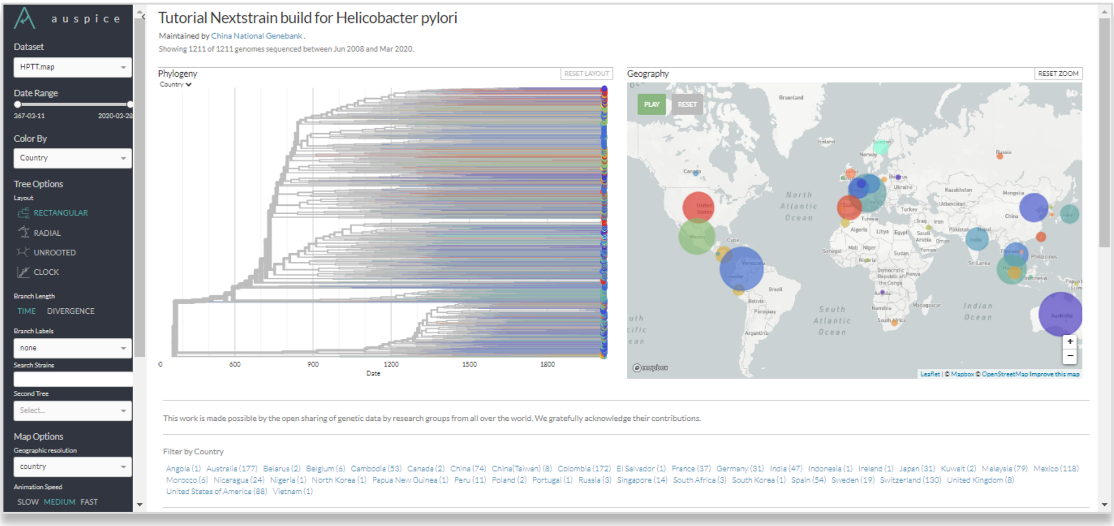

- The linking functions

When the genome matched the data in our database, it is easily to see the geographic location by clicking ID.

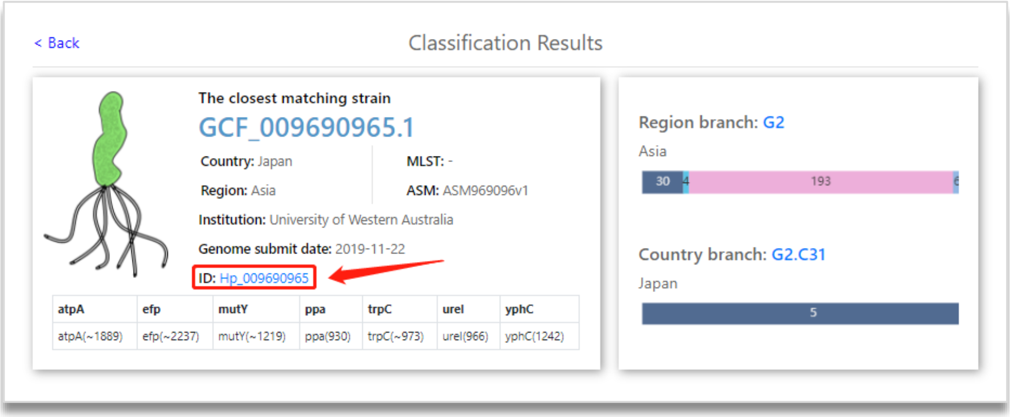

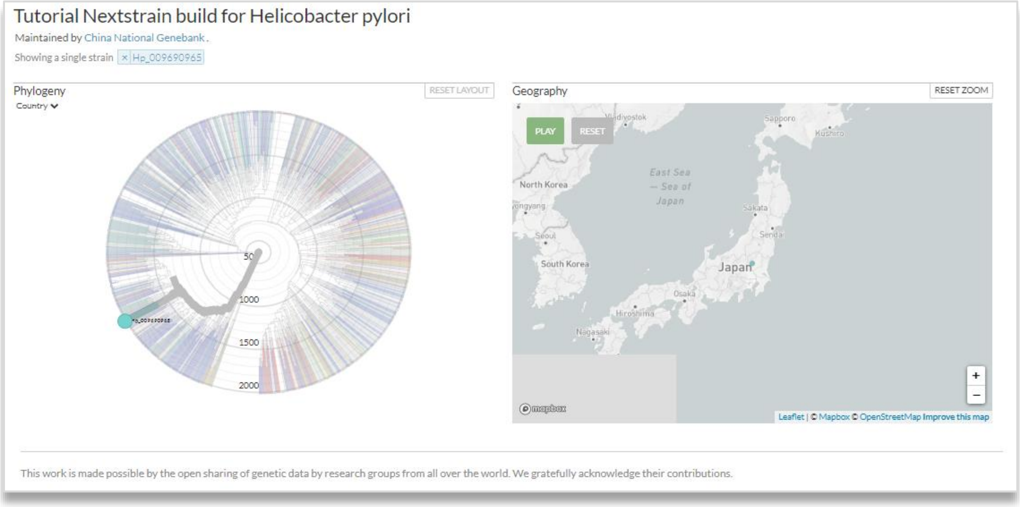

When the genome matched the data in our database, it is easily to see the phylogenetic relationship and geographic location by clicking the group ID (the letters in blue).

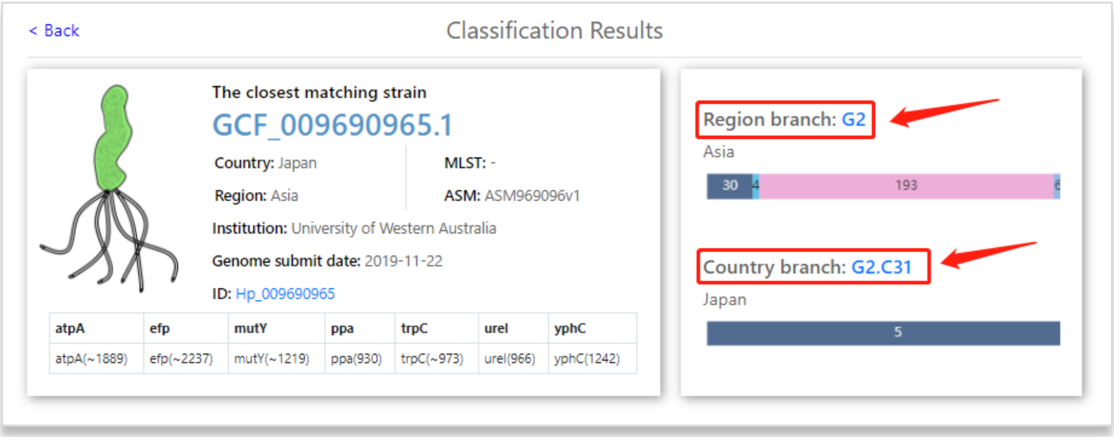

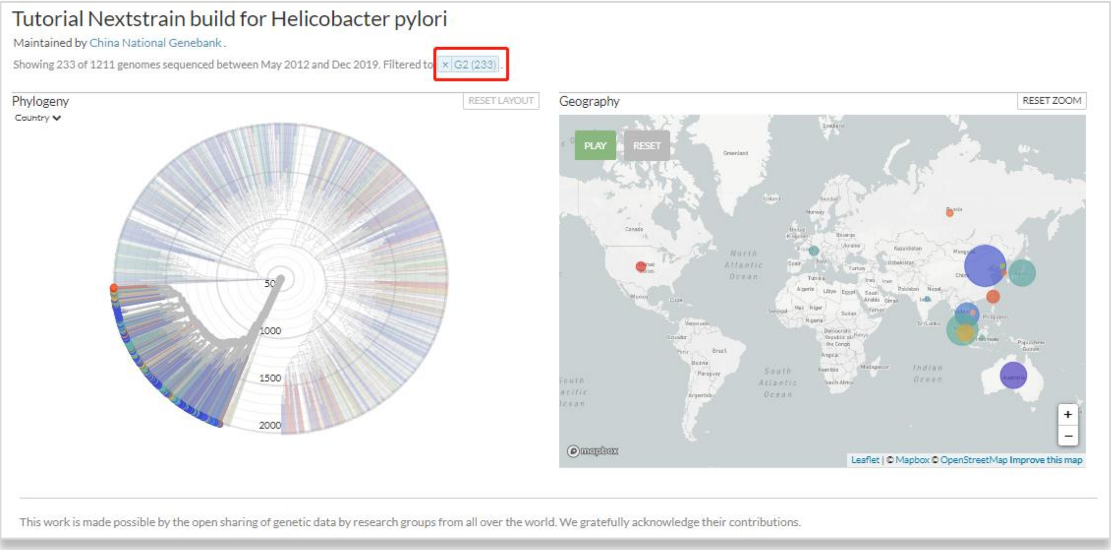
Tutorial Nextstrain build for Helicobacter pylori
Maintained by China National Genebank.
Showing 233 of 1211 genomes sequenced between May 2012 and Dec 2019. Filtered to × G2 (233).
Phylogeny
RESET LAYOUT
Country
Geography
RESET ZOOM
PLAY
RESET
Leaflet | © Mapbox © OpenStreetMap Improve this map
This work is made possible by the open sharing of genetic data by research groups from all over the world. We gratefully acknowledge their contributions.

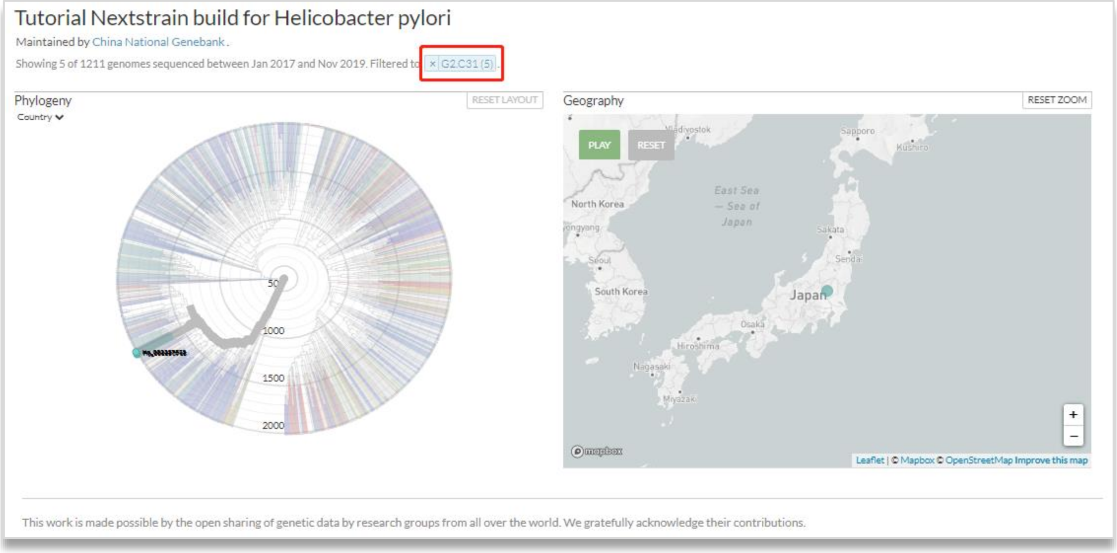
Tutorial Nextstrain build for Helicobacter pylori
Maintained by China National Genebank.
Showing 5 of 1211 genomes sequenced between Jan 2017 and Nov 2019. Filtered to × G2.C31 (5).
Phylogeny
RESET LAYOUT
Country
Geography
RESET ZOOM
PLAY
RESET
North Korea
South Korea
Japan
East Sea — Sea of Japan
Leaflet | © Mapbox © OpenStreetMap Improve this map
This work is made possible by the open sharing of genetic data by research groups from all over the world. We gratefully acknowledge their contributions.
